# Humoral Immune Correlates Analysis of Four Vaccines Against SARS-CoV-2 in Rhesus Macaques

**DOI:** 10.21203/rs.3.rs-9404931/v1

**Published:** 2026-04-30

**Authors:** Michael P. Fay, Allyson Mateja, Chris Cirimotich, Jennifer Garver, Wantong Du, Michael Anderson, April Brys, Carol Sabourin, James Long, James Little, Sara Duncan, Jason Mott, Tanima Sinha, Nina Malkovich, Gregory V. Stark, Daniel C. Sanford

**Affiliations:** 1 NIH Biostatistics Research Branch/DCR/NIAID, Rockville, MD, USA.; 2 Clinical Monitoring Research Program Directorate, Frederick National Laboratory for Cancer Research, Frederick, MD, USA.; 3 Battelle Memorial Institute, Columbus OH, USA.; 4 Biomedical Advanced Research and Development Authority (BARDA), Administration for Strategic Preparedness and Response, U.S. Department of Health and Human Services, Washington, DC, USA.; 5 Tunnell, Contractor Supporting BARDA, Berwyn, PA, USA.

## Abstract

We assessed correlates of protection (COP) in a vaccine trial of 126 Rhesus macaques randomized to placebo or one of four vaccines (mRNA-1273 [Moderna], Ad26.COV2.S [Janssen], NVX-CoV2373 [Novavax], and CoV2 preS dTM+AS03 [Sanofi]) at varying doses and challenged with SARS-CoV-2 (USA-WA1/2020 strain). Four immune marker assay readouts (live virus microneutralization assay, Meso scale discovery electrochemiluminescence immunoassay, and pseudovirus neutralizing assay using 50% or 80% inhibitory dose) measured at two time points (2 weeks prior to challenge and day of challenge) were assessed as correlates of protection. Protection was defined as ability to predict reduction in one of 12 measures of viral load (two RT-qPCR assays [subgenomic (E protein) and genomic (N1)], two time periods [two days post challenge and area under the curve (AUC) from challenge to the last day of sample collection], and three locations [nasal swab, oropharyngeal swab, and bronchioalveolar lavage]). There was high correlation among all immune marker measurements (all correlations ≥0.86) and viral load measurements (all correlations≥0.87). In 28 of 96 models predicting viral load from an immune marker, adjusted R-squared was ≥0.4 (maximum=0.52). In most of those 28 models, viral load was measured using nasal swab and summarized by AUC. In 56% of cases, a simple model predicting viral load from an immune marker explained >80% of variability compared to models that additionally contained dose parameters for different vaccines. Further, the observed viral load for animals receiving each vaccine showed good agreement to their predicted values modelled only using data from animals receiving the other three vaccines (agreement coefficients between 0.25–0.59). These analyses suggest that neutralizing titers and antibody binding levels are correlates of protection that predict reduction in viral load across these four different vaccines.

## Introduction

During the COVID-19 pandemic of 2020 lasting through 2023, Operation Warp Speed (OWS) funded several vaccines that completed testing in animal models and Phase 1–3 clinical trials at record-setting speeds. In the context of a pandemic, this multi-parallel approach was justified by the potential for rapid vaccine development and the global need for a robust vaccine supply chain. Large placebo-controlled clinical studies showed several vaccines had high vaccine efficacy. Vaccine administration under Emergency Use Authorizations (EUAs) for OWS funded vaccines began as early as December 2020 for the Moderna mRNA-1273 vaccine[1], with distribution of the Janssen Ad26.COV2.S vaccine beginning a few months later[2, 3]. The Novavax NVX-CoV2373 vaccine subsequently received an EUA on July 13, 2022[4, 5] and the Sanofi CoV2 preS dTM+AS03 vaccine and bivalent derivates against novel variants has continued to be developed clinically[6–8]. However, even as these first-generation vaccines were initially distributed, evidence of waning immunity and the emergence of Variants of Concern (VOCs) suggested a need for next-generation vaccines or alternative vaccination strategies to improve protection against VOCs, provide protection against multiple variants of SARS-CoV-2, or prolong the anamnestic response [9–12]. Due to the high rate of protection against severe disease and death attributed to each of these vaccines[13–16], these products represent a logical starting point for use in the development of next-generation vaccines.

Correlates of protection (COP) are clinical markers that can be measured post-vaccination to predict immunologic protection. There have been extensive efforts to characterize COP for SARS-CoV-2 related protection against infection, protection against disease, and discrimination between protection of mucosal versus systemic infection as reviewed by Goldblatt *et al*.[9]. While these data are instrumental to understanding the complex interplay between SARS-CoV-2 and humans, evaluating new candidate vaccines through placebo-controlled clinical trials is no longer practical given the high degree of previous immunologic exposure in the global population (either by SARS-CoV-2 infection or prior vaccination)[17–19]. One alternate approach to assessing efficacy and durability of the immunologic responses generated by vaccines is testing candidate products in the rhesus macaque (*Macaca mulatta*) model of SARS-CoV-2 infection[20, 21]. Similar to humans, rhesus macaques exhibit a high degree of protection initially from neutralizing antibodies, Th1 CD4^+^ T cells, and CD8^+^ T cells following primary exposure to virus or vaccine, although titers diminish relatively quickly with subsequent protection depending on the follicular helper T cell profile[21–25]. To facilitate comparisons between immunized rhesus macaques and human clinical data, COP in rhesus macaques that correspond with efficacy in humans need to be identified. Well-defined immunologic thresholds will also accelerate vaccine development timelines by providing targets for modeling immunologic wane. Ultimately, this approach will improve understanding of breakthrough infections and reduce the post-vaccination period required to evaluate new vaccines and vaccination strategies in the nonhuman primate model. In this rhesus macaque study, vaccine efficacy is measured by one of several viral load parameters in macaques after challenge. While the data presented herein is from the original vaccines and the challenge was with the Wuhan strain, we can use this information to inform future vaccines developed for different strains.

A common characteristic of the mRNA-1273, Ad26.COV2.S, NVX-CoV2373, and CoV2 preS dTM vaccines is that each elicits immunity against the SARS-CoV-2 spike (S) glycoprotein [7, 26–28]. However, each vaccine was developed with unique scientific approaches and formulations that result in variation between the exact immune responses generated. To briefly summarize, mRNA-1273 consists of a nucleoside-modified transcript for the SARS-CoV-2 glycoprotein with transmembrane anchor (S-2P antigen) stabilized in its prefusion conformation[26]. The vaccine utilizes a lipid nanoparticle delivery system previously developed for other viral vaccines[29, 30] and the nucleoside modifications prevent early activation of interferon-associated genes[26]. The recombinant Ad26.COV2.S vaccine is a replication-deficient human adenovirus Type 26 vector expressing the full-length S protein[27]. Circumstantial timing of the Ad26.COV2.S Phase 3 clinical trial permitted efficacy comparisons against emerging VOCs, which demonstrated variability in neutralizing antibody activity but consistent protection by cellular responses [12, 31]. NVX-CoV2373 and CoV2 preS dTM are both adjuvanted recombinant protein vaccines although there are differences between the recombinant proteins and adjuvants used. NVX-CoV2373 contains 5 μg of trimeric full-length S glycoproteins of the Wuhan-Hu-1 strain derived from a baculovirus expression vector system and is adjuvanted by 50 μg of Matrix-M[13, 28]. The CoV2 preS dTM recombinant S protein is derived from a baculovirus expression vector system and the vaccine contains stabilized prefusion S protein adjuvanted by the oil-in-water emulsion AS03 system[7, 32].

As each of these vaccines have been extensively characterized for safety and efficacy in clinical trials, attempting to make merit comparisons between these products based on laboratory studies would be of little value. However, the variations in immune responses generated by each of these vaccines present a novel opportunity to identify common COP against SARS-CoV-2 infection in a product-agnostic animal model of breakthrough infections. Here, we investigated SARS-CoV-2 breakthrough infections in the rhesus macaque model following immunization with the mRNA-1273, Ad26.COV2.S, NVX-CoV2373, or CoV2 preS dTM vaccine. By conducting parallel analysis of each vaccine and simultaneously testing multiple subclinical immunization doses, we demonstrate a model of waning immunity for the identification of COP that may translate to efficacy in humans and accelerate vaccine development timelines. The lower vaccine doses, including some that induce a sub-optimal immune response, included in our study substantially reduced time-to-breakthrough following vaccination and allowed for further characterization of rhesus macaque COP.

The four vaccines have been studied previously in nonhuman primates: mRNA-1273 (Moderna) [23] [33], Ad26.COV2.S (Janssen) [22], NVX-CoV2373 (Novavax) [34], and CoV2 preS dTM+AS03 (Sanofi) [35]). Although correlate of protection analyses have been performed on individual vaccines (see e.g.,[33]), no previous correlates of protection study has combined these four vaccines. The novelty of this approach is that by design and randomization, all four vaccines are tested at multiple dosages in the same study, and thus importantly, on the same population of animals, with the same challenge dose, by the same SARS-CoV-2 strain, at the same facility, with the same assays, and the same timing of the assessments of immune markers and viral loads after challenge.

We examined eight single immune markers (four assays measured at two time points) and 12 viral load measurements (two assays for two time periods and three locations each). Our goal was to assess the ability of immune markers to predict viral load post-challenge using correlates of risk (CoR) analyses. For this study CoR are biomarkers associated with viral load instead of clinical sign of disease because SARS-CoV-2 typically leads to only mild clinical disease in Rhesus macaques [36]. COP are biomarkers causally related to vaccine efficacy [37], and here we define vaccine efficacy using the expected ratio of viral measurements in vaccinated NHP relative to control NHP.

We began by studying how correlated the immune markers are to each other, and we did the same for the viral load measurements. We fit models of the dose effects within each vaccine on both the immune markers and the viral load, to ensure that both have a causal relationship to the vaccines. Then by cross validation, we repeatedly chose, from several classes of models, the model that best predicts each viral load measurement using each of the immune markers. These models did not use vaccine or dose. However, to measure how much the vaccine and dose matters, we estimated the percent of variance explained by the immune marker alone compared to the model with the immune marker plus a separate dose effect for each vaccine. Furthermore, we repeatedly used data from three of the vaccines to estimate a model including an immune marker, then used the resulting model to predict viral load using immune marker data from animals that received the vaccine not included in the model fit. The agreement of the modeled viral load to the observed viral load was determined with a concordance correlation coefficient. Finally, we plotted a model of vaccine efficacy by immune marker allowing a separate model for each vaccine to visually compare the relationship between the four vaccines.

## Methods

### Study Design

**Figure 1.**
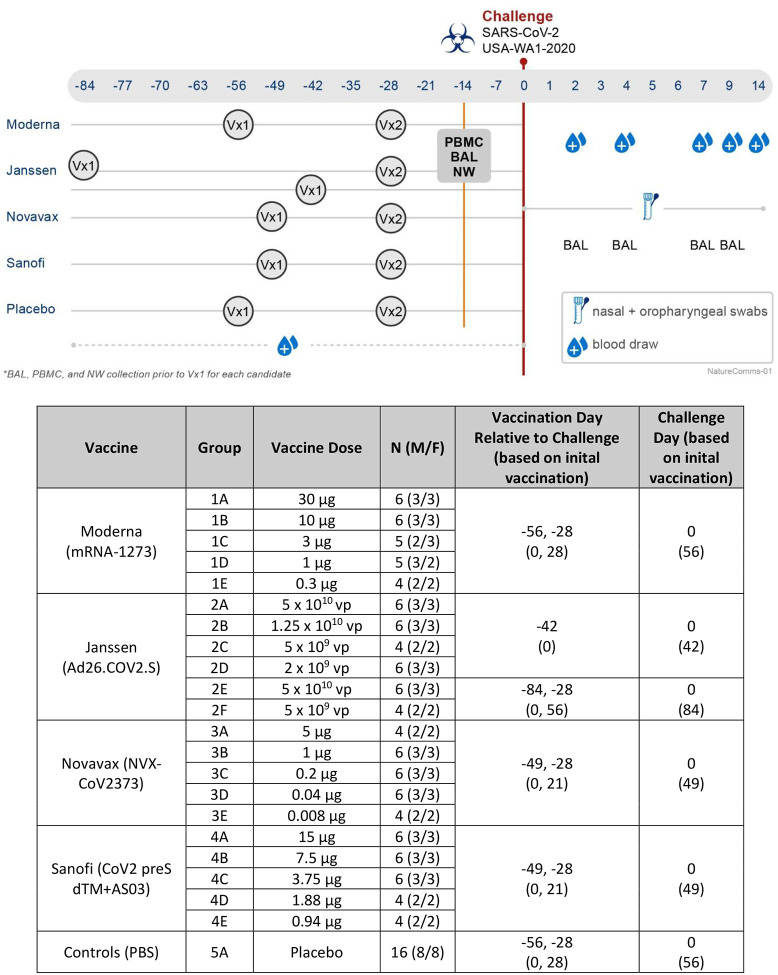
Study Design Summary. Timeline for study by vaccine. Vaccinations depicted in circles (Vx1, Vx2). BAL = bronchoalveolar lavage. PBMC = peripheral blood mononuclear cells. NW = nasal wash. Pre-challenge (during the vaccination portion), bloods were collected prior to the first vaccination, and then weekly until the day of challenge; BAL, NW, and PBMCs were collected prior to the first vaccination and 14 days prior to challenge. Post-challenge, blood was collected on Days 2, 4, 7, 9, and 14; BAL was collected on Days 2, 4, 7, and 9; and nasal and oropharyngeal swabs were collected on the day of challenge (prior to challenge), Days 1 through 7, 9, and 14.

Battelle is a Public Health Service (PHS) Animal Welfare Assurance approved facility. The study protocol was approved by the Battelle Institutional Animal Care and Use Committee (IACUC) and the study performed at Battelle was conducted under United States Food and Drug Administration’s (FDA) Good Laboratory Practice (GLP) Regulations (21 CFR Part 58). All aspects of the animal study protocol were designed to minimize stress in the animals. A total of 126 (63 males and 63 females) naïve rhesus macaques of Chinese origin, hereafter referred to as nonhuman primates (NHPs), weighing 3.40 to 10.27 kg, and being >2.5 years of age were used. Animals were stratified by body weight and sex and first randomized into either placebo (n=16) or one of the four vaccine arms, animals were then randomized into five or six dose levels within each active vaccine group and challenged on one of eight different challenge days such that NHPs from each vaccine or the placebo group were included in each challenge day. NHPs were immunized at different times and doses with either the Moderna mRNA1273 vaccine (as two dose regimen on Days 0 and 28), the Janssen Ad26.CoV2.S vaccine (as single dose or two dose regimen on Days 0 and 56), the Novavax NVX-CoV2373 vaccine (as two dose regime on Days 0 and 21), or the Sanofi CoV2 preS dTM+AS03 vaccine (as two dose regimen on Days 0 and 21). Additionally, a placebo group received saline solution on Days 0 and 28. Twenty-eight days after the second vaccine dose (or 42 days after the single dose Ad26.CoV2.S) all animals were challenged (see Figure 1). Sample collection schedules were the same for all arms post-challenge (Figure 1).

### Serum Collections

Blood samples were taken from a femoral artery or vein and collected into serum separator tubes (SSTs), processed to serum, aliquoted and stored at ≤−80°C until analysis. Prior to challenge (during the vaccination portion of the study) blood was collected weekly from baseline (prior to first vaccination) through the day of challenge. Post-challenge, blood was collected on Days 2, 4, 7, 9, and 14 (see Figure 1).

### BAL and Nasal Wash

Bronchoalveolar lavage (BAL) sampling was performed while the animal was anesthetized. Briefly, animals were intubated with an appropriately sized endotracheal tube, and a sterile feeding tube was inserted into the lumen of the endotracheal tube. Sterile PBS was administered (up to three times per collection, depending on how productive the VAL collection was) through the feeding tube at a dose of ~3–5 mL/kg, and the BAL fluid retrieved by gentle suction on the feeding tube with a syringe. Pre-challenge, BAL samples occurred at baseline (prior to vaccination), and fourteen days prior to challenge (see Figure 1). BAL collected pre-challenge was processed (centrifuged at 500 rfc for 5 min at 4° C to remove cells) and the supernatant split into 4 aliquots and stored at ≤−80° C. BAL collected post-challenge were not centrifuged to remove cells, but split into 4 aliquots and stored at ≤−80° C.

Nasal washes were performed at the same timepoints as pre-challenge BALs. Nasal wash samples were collected by flushing the nares of sedated NHPs with sterile PBS (0.5 ml per nare), aliquots were made and stored at ≤−80° C.

### Systems Serology

Serum samples collected weekly prior to challenge were stored at ≤−80°C until shipped for systems serology analyses at the Ragon Institute. In addition, nasal wash and BAL collections at baseline (prior to vaccination) and fourteen days prior to challenge were also shipped for analysis (presented separately, manuscript in preparation).

### Peripheral Blood Mononuclear Cells (PBMCs)

Whole blood for isolation of peripheral blood mononuclear cells (PBMCs) was collected in cell preparation tubes (BD Vacutainer CPT tubes, Becton Dickinson and Company BD, Franklin Lakes, NJ) at baseline (prior to vaccination) and fourteen days prior to challenge (see Figure 1). After isolation, PBMC specimens were washed, counted using a Guava Easycyte and ViaCount, and aliquots prepared at a target concentration of 1 × 10^7^ viable cells/vial. PBMCs were cryopreserved in CryoStorCS solution. The cryovial tubes were transferred into a chilled cryofreezing container filled with 100% isopropanol and placed in a freezer set to maintain ≤ −60 °C. After a minimum of 12 hours at ≤ −60°C, the cryovial tubes were transferred to a liquid nitrogen storage tank. PBMCs were shipped offsite for analysis (results presented separately, manuscript in preparation).

### Nasal and Oropharyngeal Swab Collections

Samples were collected on the day of challenge (prior to challenge) and on Days 1 through 7, 9, and 14 post-challenge (see Figure 1). Samples were aliquoted and stored at ≤−80° C until analysis.

### Microneutralization (MN) Assay

Two-fold serial dilutions of heat-inactivated serum samples were pre-incubated with virus for 60 minutes at 37°C ± 2°C. The virus/serum mixture was then added to a 90 to 100% confluent monolayer of Vero E6 cells (BEI, Cat. No. NR-596) in 96-well plates and incubated for 43 ± 3 hours at 37°C ± 2°C with 5% CO_2_. Following incubation, the inoculum was removed, and monolayers were incubated for 30 minutes in 80% cold acetone to allow cell fixation. Plates were incubated with anti-nucleocapsid protein primary antibody cocktail (clones HM1056 and HM1057) (EastCoast Bio, North Berwick, ME) for 60 ± 5 minutes at 37°C ± 2°C. The plates were washed and the secondary antibody (goat anti-mouse IgG Horse Radish Peroxidase (HRP) conjugate; Fitzgerald, North Acton, MA) was added to the wells and the plates were incubated for 60 ± 5 minutes at 37°C ± 2°C. After the plates were washed, the substrate was added, and the plates were incubated at 37°C ± 2°C. Stop solution was added and the plates were read for optical density at 405 nm wavelength. Neutralizing activity was defined as at least 50% reduction in signal from the virus only (VC) wells relative to cells control (CC) wells following the formula [(average VC –average CC)/2] + average CC. The median neutralizing titer (MN_50_) was calculated using Spearman-Kärber analysis method.

MN_50_ results were log base 10-transformed for the analysis. MN_50_ values less than the limit of detection (LOD) of 44.22 were considered negative for qualitative summaries and replaced with one-half the LOD (22.11) for quantitative calculations. In addition, for MN_50_ values greater than or equal to the LOD and less than the LLOQ (109.98), one-half of the LLOQ (54.99) was used in the quantitative calculations. This assay was validated at Battelle for NHP.

### Meso Scale Discovery Electrochemiluminescence Immunoassay (MSD-ECLIA)

The capacity of NHP serum IgG antibodies to bind SARS-CoV-2 spike protein was assessed using the V-PLEX^®^ 96-well SARS-CoV-2 Panel 13 electrochemiluminescence immunoassay (ECLIA) for human IgG detection (K15463U) from Meso-Scale Discovery (MSD; Rockville, Maryland, USA). Each well of the ECLIA plate contained 10 unique SARS-CoV-2 spike antigens; however, the focus of this study was the wild-type (Wuhan) spike antigen that is consistent with the vaccine platform targets and challenge virus. The performance of MSD assays designed for human antibody detection with NHP serum has been shown previously and was empirically confirmed through assay optimization prior to study sample analysis [38–42].

The ECLIA was performed according to manufacturer’s instructions using reagents provided with the assay kit. ECLIA plates were blocked with Blocker A solution (150 μL per well) for approximately 30 minutes, then washed three times with MSD Wash Buffer (≥ 150 μL per well). Separately, NHP serum samples were diluted in MSD Diluent 100 in duplicate with an initial 1:500 dilution followed by serial three-fold, seven-point dilution and 50 μL of each sample dilution was applied to the blocked ECLIA plate along with the calibrator curve and provided MSD control samples. The plate was incubated at room temperature for approximately 120 ± 5 minutes with shaking at approximately 700 rpm and washed three times with MSD Wash Buffer (≥ 150 μL per well). Detection Antibody (50 μL per well) was applied, and plates were incubated at room temperature for 60 ± 5 minutes with shaking at approximately 700 rpm. Plates were again washed three times with MSD Wash Buffer (≥ 150 μL per well), MSD GOLD Read Buffer was applied, and the plates were incubated at room temperature for up to 22 minutes. The electrochemiluminescence (ECL) values for each well were collected using a MESO Sector S 600 microplate reader (MSD) and data were analyzed using Discovery Workbench software (version 4.0.13). The ECL measurements from each well were interpolated against the calibration curve and the final concentration in Arbitrary Units per milliliter (AU/mL) at each dilution point was assigned by multiplying the result by the dilution. The two AU/mL concentrations at each dilution point were averaged and the final reportable value for a given test sample was the average of the acceptable AU/mL concentrations across the dilution range for the sample. While this kit could assess binding to 10 different SARS-CoV-2 variants, only the results from the Wuhan (wild type) SARS-CoV-2 spike protein antigen are reported.

Wuhan antigen results were log base 10-transformed for the analysis. Reportable value units are AU/mL. Values reported as “<LOD” (less than the limit of detection) were considered negative for qualitative summaries, and values reported as “<LLOQ” (less than the lower limit of quantitation) were considered positive for qualitative summaries. For values reported as “<LOD”, one-half of the LOD (547 AU/mL), 273.5 AU/mL, was used in the quantitative calculations. For values reported as “<LLOQ”, one-half of the LLOQ (855 AU/mL), 427.5 AU/mL, was used in the quantitative calculations. This assay was validated at Battelle for NHP.

### Pseudovirus Neutralization Assay (PsVNA)

The capacity of NHP serum to neutralize SARS-CoV-2 infection was evaluated using infection of a lentivirus vector pseudotyped with SARS-CoV-2 spike protein (D614G variant) in 293T/ACE2 cells (cell line kindly provided by Drs. Mike Farzan and Huihui Ma at The Scripps Research Institute, San Diego). Methods and materials were transferred from the laboratory of Dr. David Montefiori at the Duke Human Vaccine Institute. Briefly, heat-inactivated NHP serum samples were diluted in growth medium (DMEM supplemented with 10% FBS, 25mM HEPES, and 50 μg/mL Gentamicin) in duplicate with an initial dilution of 1:10 followed by serial five-fold, seven-point dilution in 96-well plates. Serum dilutions (100 μL) were mixed with 50 μL of pseudotyped lentivirus (PsV) and mixtures were incubated for 45–90 minutes at 37° C ± 2° C [43]. The quantity of PsV used per reaction was empirically determined to target an appropriate RLU range based on assay performance at Battelle. A suspension of 293T/ACE2 cells (100 μL at 100,000 cells/mL) was added and the mixture was incubated at 37° C ± 2° C for 72 ± 1 hours. Following this incubation, supernatant was removed, the cells were lysed with Cell Lysis Buffer (Promega; Madison, Wisconsin, USA), and Bright-Glo Luciferase reagent (Promega) was added. The levels of luminescence were determined in relative light units (RLU) using a GloMax^®^ Navigator luminometer (Promega). The percent neutralization was determined by calculating the difference in average RLU between virus control (cells plus virus, no serum) and test sample dilutions, dividing this result by the average background-subtracted RLU of the virus control, and multiplying by 100. The 50% and 80% inhibitory doses (ID_50_ and ID_80_, respectively) were expressed as the reciprocal of the serum dilution required to reduce RLU by 50% and 80%, respectively.

ID_50_ and ID_80_ results were log base 10-transformed for the analysis. ID_50_ and ID_80_ values less than the LOD (461 for ID_50_ and 43 for ID_80_) were considered negative for qualitative summaries. For ID_50_ and ID_80_ values less than the LOD, one-half of the LOD (230.5 for ID_50_ and 21.5 for ID_80_) was used in the quantitative calculations. In addition, for ID_50_ and ID_80_ values greater than or equal to the LOD and less than the LLOQ (506 for ID_50_ and 63 for ID_80_), one-half of the LLOQ (253 for ID_50_ and 31.5 for ID_80_) was used in the quantitative calculations. This assay was validated at Battelle for NHP.

### Challenge

On the day of challenge, NHPs were anesthetized and challenged with SARS-CoV-2 (USA-WA1/2020) via the intratracheal (0.5 mL) and intranasal (0.25 mL per nostril) routes with a dose of 6.56 × 10^5^ TCID_50_. Briefly, the animal was intubated with an appropriately sized endotracheal tube, placed in dorsal recumbency, and 0.5 mL of challenge material administered intratracheally. While still in dorsal recumbency, the animal was then challenged with material by the intranasal route.

### RT-qPCR Nucleocapsid Protein (N1) Genomic Analysis

BALs, nasal swabs, and oropharyngeal swabs were analyzed by RT-qPCR. RNA was isolated using the Indispin QIAcube HT Pathogen Kit (Indical Bioscience, Germany) on the QIAcube HT instrument (Qiagen, Germany). The isolated RNA was then evaluated in RT-qPCR using the TaqMan Fast Virus 1-step Master Mix (Thermo Fisher Scientific) on a QuantStudio Flex 6 Real-Time PCR System (Applied Biosystems; Foster City, CA). The primers and probe were specific to the SARS-CoV-2 nucleocapsid protein gene, corresponding to the N1 sequences from the Centers for Disease Control and Prevention (CDC) 2019-Novel Coronavirus (2019-nCoV) Real-Time RT-PCR Diagnostic Panel (https://www.cdc.gov/coronavirus/2019-ncov/lab/rt-pcr-panel-primer-probes.html) except that the probe quencher was modified to Non-Fluorescent Quencher-Minor Groove Binder (NFQ-MGB) (Thermo Fisher Scientific). A standard curve comprised of synthetic RNA (see supplement for specific sequence) containing the target sequence from SARS-CoV-2 isolate WA1 sequence (GenBank Accession Number MN985325.1) (Bio-Synthesis, Inc.; Lewisville, TX) was included on each PCR plate for absolute quantitation of SARS-CoV-2 RNA copies in each sample. Thermocycling conditions were as follows: Stage 1 - 50°C for 5 min for one cycle; Stage 2 - 95°C for 20 sec for one cycle; Stage 3 - 95°C for 3 sec and 60°C for 30 sec for 40 cycles. Data analysis was performed using the QuantStudio 6 software-generated values (total copies per well of each sample) and additional calculations to determine SARS-CoV-2 N1 copies per mL of fluid. This assay was validated at Battelle for NHP.

### RT-qPCR Envelope Protein (E) Subgenomic Analysis

Following isolation and evaluation using the N1 genomic assay, the isolated RNA was then evaluated as described above using primers and probes specific to the SARS-CoV-2 E gene sequences [44], and the reverse primer and probe sequences previously described [45] (Integrated DNA Technologies, Iowa). A standard curve comprised of synthetic RNA (see supplement for specific sequence) containing the target sequence from SARS-CoV-2 isolate WA1 sequence (GenBank Accession Number MN985325.1) (Bio-Synthesis, Inc.; Lewisville, TX) was included on each PCR plate for absolute quantitation of SARS-CoV-2 copies in each sample. Thermocycling conditions were as follows: Stage 1 - 50°C for 5 min for one cycle; Stage 2 - 95°C for 20 sec for one cycle; Stage 3 - 95°C for 3 sec and 60°C for 30 sec for 40 cycles. Data analysis was performed using the QuantStudio 6 software-generated values (total copies per well of each sample) and additional calculations to determine SARS-CoV-2 E gene subgenomic (Esg) RNA copies per mL of fluid. This assay was validated at Battelle for NHP.

### Limits and AUC for RT-qPCR data

The reported “<LOD” (less than the limit of detection) and the reported “<LLOQ” (less than the lower limit of quantitation), were considered negative and positive results, respectively, in qualitative analysis and half of the corresponding LOD or LLOQ were used in quantitative analysis. For Nucleocapsid Gene Segment N1 assay, the LOD was 230 gene copies/mL (gc/mL) and the LLOQ was 2,224 gc/mL and for Small Envelope Protein E Gene Subgenomic RNA assay, the LOD was 422 gc/mL and the LLOQ was 3,881 gc/mL. RT-qPCR results were log base 10-transformed for the analysis.

Areas under the curve (AUC) were calculated using the trapezoidal rule for each log base-10 transformed viral burden endpoint. For nasal swabs and OP swabs samples, AUC were calculated from Day 0 to Day 14. For BAL samples, AUC were calculated from Day 0 to Day 9 and half of the LOD was used in the calculations for Day 0. BAL samples were not collected on the day of challenge as the sampling collection is more invasive and could interfere with the challenge, therefore, the values reported on Study Day -14 were used as the Day 0 measurements (all pre-challenge PCR values were reported as <LOD). To compute the AUC, the area under the line connecting reported viral burden values from two consecutive sampling time points was calculated. The areas for each of these trapezoids were then summed across all pairs of consecutive time points for the animal, as shown by the equation below:

$$AUC=\sum_{i=1}^{T-1}\;\frac{{\text{log}}_{10}\left(x_{i}\right)+{\text{log}}_{10}\left(x_{i+1}\right)}{2}\times \left(d_{i+1}-d_{i}\right)$$

where *T* is the total number of sampling time points for the animal, *x*_*i*_ is the reported viral burden value at the *i*^*th*^ time point, and *d*_*i*_ is the Study Day of the *i*^*th*^ time point. The mean and 95% confidence interval, standard deviation, minimum and maximum, and number of animals were calculated for each group. Time course plots of the means and 95% confidence intervals were prepared.

### Statistical analysis on Correlates of Risk and Protection

All analyses in this paper were pre-defined in the statistical analysis plan (SAP, see Supplement), except when noted. Confidence intervals for Spearman correlations used asymptotic normality of Fisher’s Z transformation [46]. Immune markers used log10 transformations with values less than the LOD set to one-half the LOD. Confidence intervals used one-sample t-test methods on the log10 transformed values. We defined the dose score as 0 for placebo, 1 for the minimum dose for each vaccine, 2 for the dose 10 times larger than the minimum, and generally, 1 plus log10 of the ratio of the dose to the minimum (see e.g., Supplemental Figure 5). Models of dose score by viral load used linear generalized estimating equations (GEE) clustered by calendar day of challenge using working independence correlations with confidence intervals by Wald methods using the default small sample adjustment in the saws R package version 0.9–7.0 [47] [48]. These models allow a different effect for each vaccine by estimating a different adjustment factor for each vaccine’s dose score, but include a common intercept for an unvaccinated animal estimated from the response observed in the common, unvaccinated control group.

For each of 96 (12 viral load measurements by 8 immune marker measurements) pairs of viral load/immune marker the best fit model was selected by cross validation from 5 classes of models (linear, exponential, segmented line regression, natural cubic splines, and 4-parameter logistic). In the cross-validation procedure, 10% of the data was left out as the test set, stratified by challenge day, and the remaining 90% of the data was fit to each class of model. These fitted models were used to calculate predicted viral load for the test set, and the adjusted R^2^ was calculated for each model class. We recorded which model type had the highest adjusted R^2^ and repeated this procedure 500 times. The model class with the best fit was selected as the one selected the most times out of the 500. After the model class was selected, the confidence interval was calculated assuming that the selected model class was correctly specified, ignoring the variability of the selection procedure. The coverage of this procedure was found to be reasonable by extensive simulations done prior to receiving the data from the study (see Supplement).

The percent of variance explained is defined as:

$$PVE=100\times \frac{R_{a}^{2}(\text{reference})}{R_{a}^{2}(\text{reference}+\text{extra})}$$

where $R_{a}^{2}(\text{reference})$ represents the adjusted R-squared for the reference model (e.g., the model that only includes the immune marker for prediction as selected by cross-validation), and $R_{a}^{2}(\text{reference}+\text{extra})$ represents the adjusted R-squared for the model that adds extra variables [49]. Percent of variability explained (PVE) values greater than 100% were set to 100%. PVE of 100% indicates that the simpler reference model explains at least as much of the variance as the more complicated model does after adjusting for the number of parameters in the model. Confidence intervals for the PVE were calculated using nonparametric bootstrap methods [50]. The “reference + extra” model included the immune marker (reference) plus parameters that allow a different slope of the linear predictor part of the model (linear or exponential) for each vaccine type. This latter model was a modification to one suggested in the SAP (see supplement for details).

To explore using different vaccines to model and predict viral load responses, we repeatedly fit the best model class selected by cross validation after leaving out one of the four vaccine types. Using that fit we predicted the viral load response for the animals given the vaccine that was left out. We measured agreement of the model with the observed viral load using the concordance correlation (random marginal agreement coefficient version) [51] with confidence intervals by nonparametric bootstrap [50]. Concordance correlations can range from −1 to 1, with 1 denoting perfect agreement, 0 denoting agreement by random chance, and −1 denoting perfect negative agreement.

We performed an exploratory analysis not pre-specified in the SAP to study COP. We picked the immune marker PsVNA ID50 | Day -14, similar to the marker Figure in Gilbert, et al. (2022), and measured viral load using AUC | Nasal Swab | subgenomic. Efficacy was measured as vaccine efficacy by viral load (VEVL), specifically, VEVL(x)=1-R(x), where R(x) is the ratio of the modelled viral load on the vaccine given the value of the immune marker is x over the mean viral load on placebo. The viral load was modelled using a linear model allowing a separate line for each vaccine. A formal causal CoP analysis was not done; however, significant positive slopes in the model of VEVL(x) suggest the immune marker plays a part in the reduction of viral load.

Results not presented are in an accompanying SAP Report (see [52]). Since the different analyses are exploratory and closely related to each other, no adjustments for multiple analyses were made.

### Data Availability

The data file and all R markdown files used to perform the analyses in this paper have been made available at https://github.com/michaelpfay/NHP_Four_SARS-CoV-2_Vaccines/ under the folder “SAP R Code Final”.

## Results

**Figure 2.**
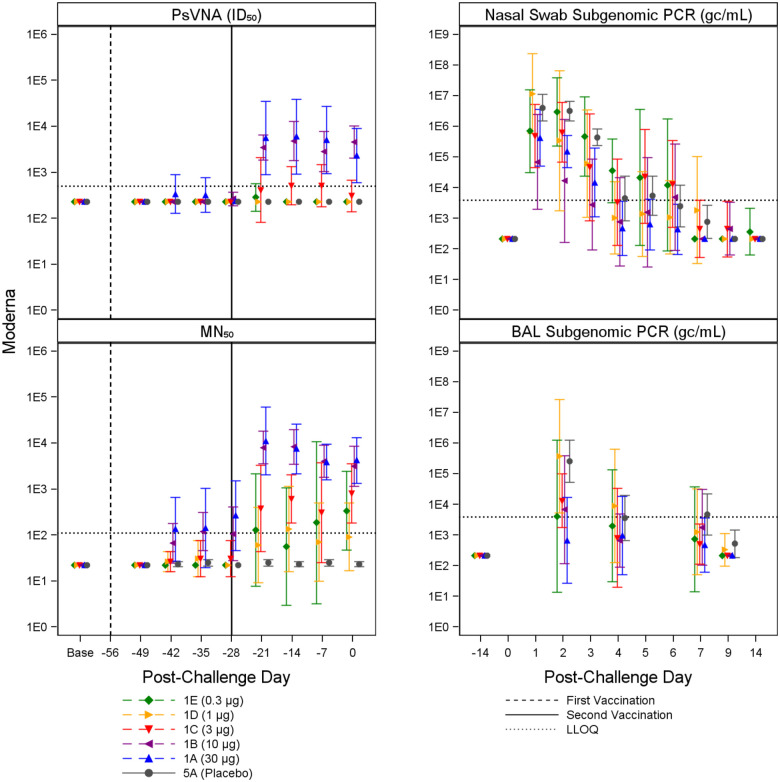
Moderna PsVNA_50_, MN_50_, and Subgenomic PCR Results. Geometric mean and associated 95% confidence intervals for PsVNA (ID_50_) and MN_50_ immune markers and BAL and nasal swab Subgenomic E PCR viral load, shown by dose and post-challenge day for Moderna and placebo (grey). Dose groups are ordered by lowest (1E, green) to highest (1A, blue). The dotted horizontal line indicates LLOQ. The first vaccination is shown with a dashed vertical line, and the second vaccination is shown with a solid vertical line.

Immune responses were observed in vaccinated animals compared to placebo controls, generally in a dose dependent manner. Animals receiving higher vaccine doses tended to have lower viral loads compared to placebo controls based on viral load assessments in the upper (nasal swab) and lower respiratory system (BAL). Figures 2 through 5 summarize the geometric mean PsVNA_50_, MN_50_, Subgenomic E nasal swab and Subgenomic E BAL PCR results by dose group for the Moderna, Jannsen, Novavax, and Sanofi vaccines, respectively. Supplemental Figures S1 through S4 summarize the geometric mean PsVNA_80_, MSD-ECLIA, and N1 nasal swab and N1 BAL PCR results by dose group for each vaccine.

### Correlations Among Immune Markers and Among Viral Load Outcomes

There was a high correlation among all pairs immune markers, but not as high among pairs viral load outcomes.

**Figure 6.**
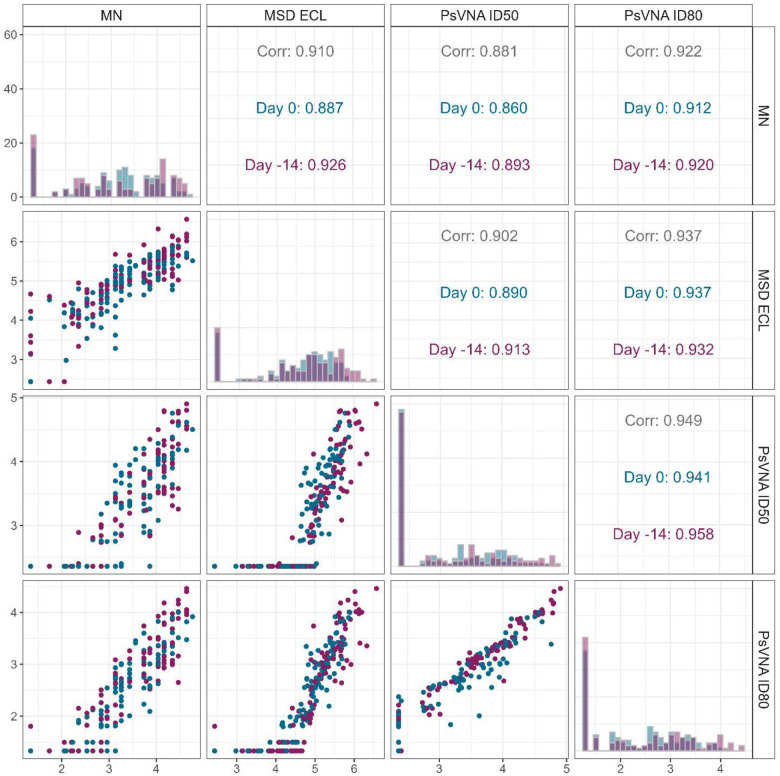
Relationship between 4 assays to measure SARS-CoV2 antibody level Meso scale discovery electrochemiluminescence immunoassay [MSD ECL], or neutralizing activity (live virus Microneutralization assay [MN], and Pseudovirus neutralizing assay using inhibitory dose of either 50% [PsVNA ID_50_] or 80% [PsVNA ID_80_]). Measurement times are challenge day (Day 0, n=126, deep blue) and 14 days prior to challenge (Day –14, n=126, maroon). Upper diagonal are Spearman correlations using all measurements (Corr), or challenge day 0 or −14. Diagonal are histograms (dark color is overlapping of the two times). All histograms have the same y-axis (shown in the upper left box). Lower diagonal are scatter plots (some dots represent multiple points). For correlations between day −14 and day 0 within assay see Table S1 (Supplement).

Figure 6 depicts the relationship between the four immune marker assays, displaying scatter plots, histograms, and Spearman correlations (for correlation 95% confidence intervals see Table S1). From the histograms, we see that the pseudovirus neutralizing assays have a higher proportion of values less than the LOD than the microneutralization and MSD-ECLIA assays. All pairwise correlations are strong (≥ 0.86), so there does not appear to be a strong statistical reason to prefer one assay over another.

**Figure 7.**
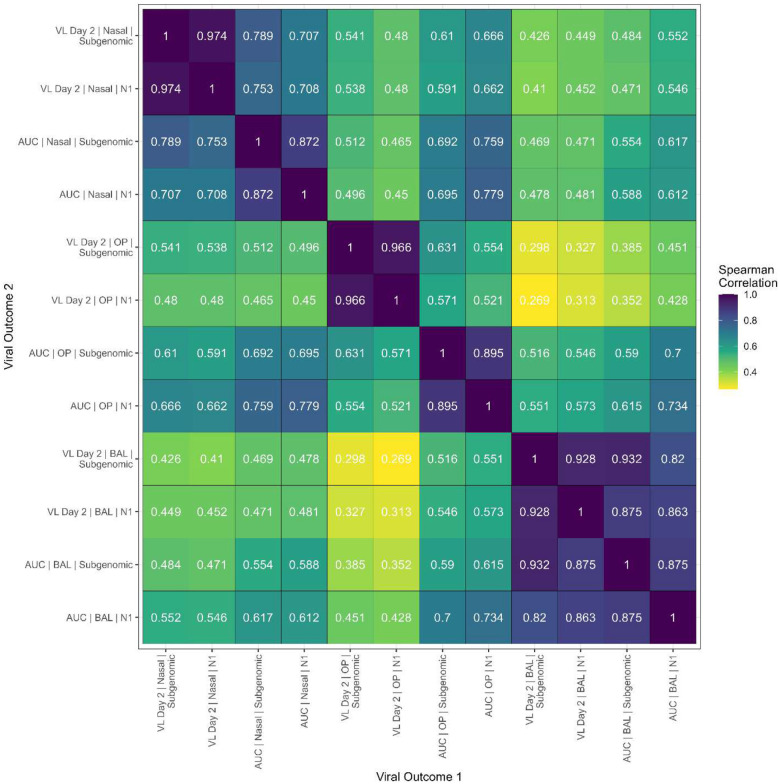
Spearman correlations between viral load (VL) measurements. There are 12 post-challenge viral load measurements for each animal: all combinations of 3 locations (nasopharyngeal swab, oropharyngeal swab, or bronchioalveolar lavage), 2 time periods (only day 2 post challenge, or area under the curve using data up to day 9 post challenge), and 2 viral assays (RT-qPCR of Nucleocapsid Gene Segment N1, RT-qPCR of the Small Envelope Protein (E) Gene Subgenomic RNA).

The story is different for the 12 viral load measurements (Figure 7, Table S2). In Figure 7, we see that within each timepoint and location pair, the two PCR assays (N1 and Subgenomic E) for measuring the viral load are highly correlated (≥ 0.87). Within each assay, the correlation between the two time periods (VL Day 2 and AUC) differs by location; there is a strong correlation for the BAL (Spearman correlations of 0.93 [Subgenomic E] and 0.86 [N1]), while there is less correlation using nasal swabs (Spearman correlations of 0.79 [Subgenomic E] and 0.71 [N1]), and an even weaker correlation using OP swabs (Spearman correlations of 0.63 [Subgenomic E] and 0.52 [N1]). Finally, the correlation between different locations is stronger with AUC (Spearman correlations between 0.55 to 0.78) than with VL Day 2 (Spearman correlations between 0.30 to 0.54).

### Assessing Vaccine Dose Response on Immune Markers for Each Vaccine

**Figure 8.**
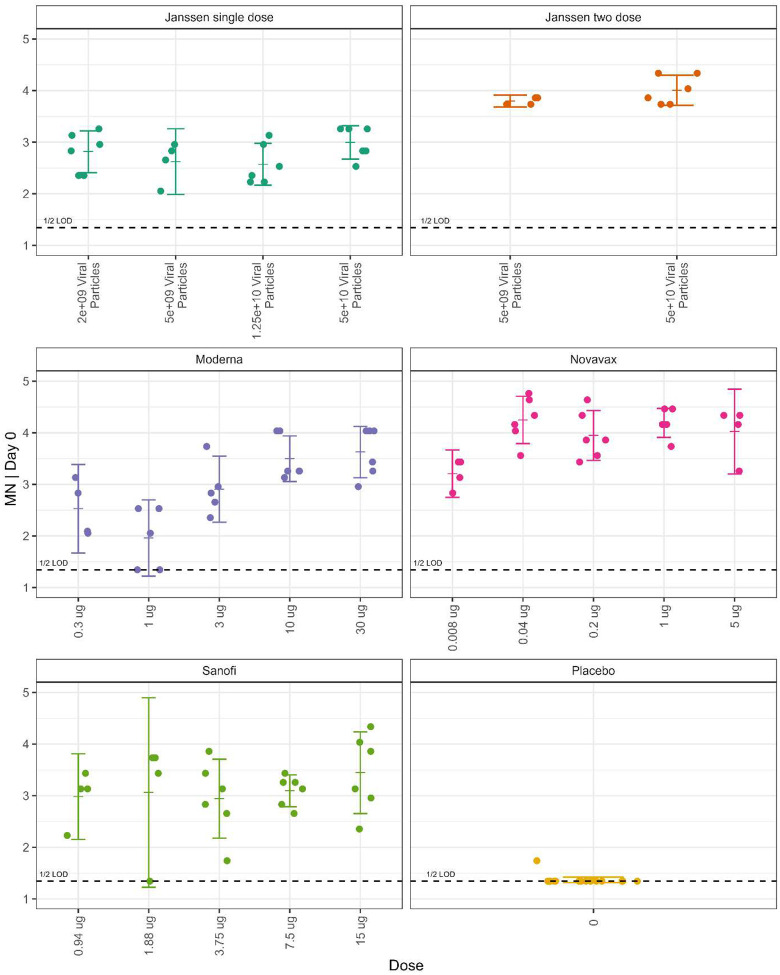
Microneutralization assay on challenge day by dose level within vaccines. Measurements are on the log10 scale [median neutralizing titer (MN50)]. Ticks are means (geometric means on original scale), with 95% confidence intervals.

**Figure 9.**
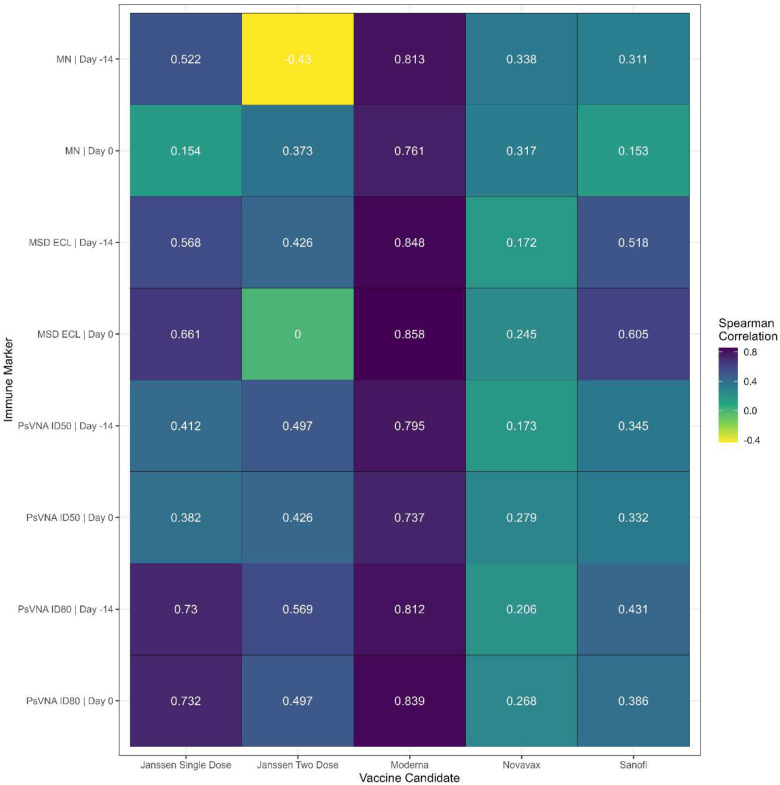
Spearman correlation of immune marker by dose for each vaccine. Janssen single dose has 4 dose levels; Janssen two dose has 2 dose levels; and Moderna, Novavax, and Sanofi have 5 dose levels each.

We next examined the relationship between dose effects and the immune markers, which differ by vaccine. Figure 8 shows the values for one immune marker, log10 MN | Day 0. Although all dose levels of the vaccines have nearly all responses greater than the limit of detection, there does not always appear to be a dose response over the dose levels within a vaccine. From this, it is difficult to infer differences between vaccines because the design of the dosage groups differs within each vaccine. The Janssen vaccine has 6 dosage groups, with four dosages using a single dose regimen and two dosages using a two-dose regimen; the dose spread from lowest dose to highest dose is a 25-fold difference for the single dose regimen, and a 10-fold difference for the two-dose regimen. The other vaccines all use two dose regimens over five doses, but the lowest dose to highest dose fold difference varies between the vaccines (Moderna has a 100-fold difference, Novavax has a 625-fold difference, and Sanofi has a 16-fold difference). In addition, the dosages differ relative to the humanized dose for each vaccine. There is a difference in how much the immune markers change as the dosage changes. Figure 9 shows the dose response by Spearman correlations between the eight immune markers (four assays by two time periods) and the dosage within vaccine. Moderna has the highest Spearman correlations, that are highly significant with the lower limit of the 95% confidence interval ranging from 0.49 to 0.71 (Table S3). The Novavax and Janssen two dose regimen have no significant Spearman correlations of the dose response on the immune markers, while the Janssen single dose regimen and Sanofi had significant correlations primarily from the MSD ECL and PsVNA ID80 immune markers.

### Assessing Vaccine Dose Response on Viral Load Outcome for Each Vaccine

We modelled dose response on viral load using a pre-specified dose score and a linear generalized estimating equation (GEE) model, and the best fit models used AUC viral load measurements.

**Figure 10.**
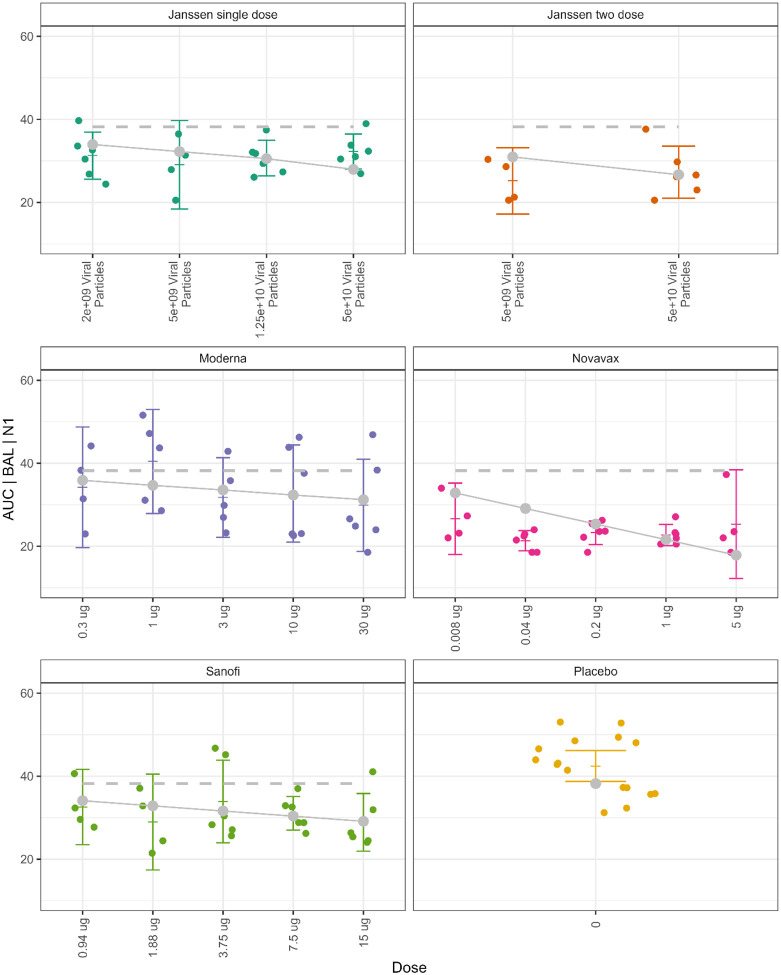
Viral load AUC|BAL|N1 (gc/mL) by vaccine dose. Colored vertical lines have tick marks for means and 95% confidence intervals. The solid gray lines and dots are the estimates from the linear generalized estimating equation model for each vaccine, where the zero-dose level is defined to be the same distance from the lowest dose as from the lowest to the next lowest dose. The modelled zero dose levels are forced to be the same as the modeled placebo level (depicted as a gray dot [placebo panel] or dotted line [all other panels]).

The dose scores are defined and depicted in Supplemental Figure S5, where the control arm has dose score 0, lowest dose within a vaccine has dose score 1, a dose 10 times higher has a dose score of 2, 100 times higher has dose score of 3, etc. (Janssen single dose groups had their doses halved before scoring in all presented analyses). We fit a separate model for each of the 12 viral load responses. We present a model with good fit in Figure 10 (estimated model parameters in Table S4), which shows the model for viral load AUC|BAL|N1. The modelled line for each vaccine has its own slope, and each line crosses the modeled placebo value at that vaccine’s zero value. The slopes for Janssen, Novavax, and Sanofi were all highly significant (all p=0.002), while the slope for Moderna was not (p=0.114). To summarize across all 12 models, first, as we would expect from the high correlation between the N1 and subgenomic assays (Figure 7), there is little difference in the significance levels between models that only differ by assay type, although the slope estimates may change, which is consistent with highly correlated assays. Second, the AUC viral load measurements using multiple time measurements provide more information about the course of infection compared to Day 2 values, therefore it is not surprising that models with AUC viral load measurements have a better fit and show more significant vaccine effects. Specifically, Day 2 measurements from the OP swab rarely showed significant vaccine effects, perhaps due to the large variability of the responses in the placebo arm.

### Prediction of Post-Challenge Outcome with Immune Correlate

To model each post-challenge viral load outcome based on an immune marker, we chose a best fit model using cross validation. Only the simple linear or exponential models were chosen and many of the fits were similar, with no particular immune marker that predicted a viral load outcome substantially better than all the others.

**Figure 11.**
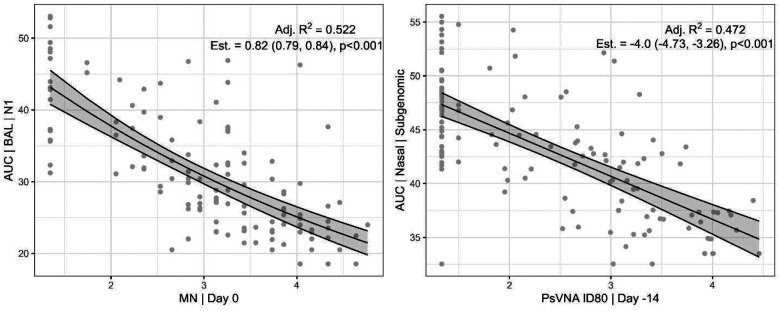
Models predicting viral load using immune markers. Models chosen by cross validation. Middle lines are the model, and the gray areas are the 95% confidence interval. (Left panel) Best fitting model by adjusted R-squared. Exponential model predicting viral load as AUC|BAL|N1 using MN|Day 0. The model estimates that viral load changes by a factor of 0.82 (95% confidence interval: 0.79, 0.84) for each 1 unit increase in the immune marker. (Right panel) Seventh best fitting model (out of 96) by adjusted R-squared. Linear model predicting viral load as AUC | Nasal | subgenomic using PsVNA ID80 | Day -14. The model estimates that viral load changes by −4.0 (95% CI: −4.73, −3.26) for each 1 unit increase in the immune marker.

For each of the 96 (12 viral load measurements by 8 immune marker measurements) pairs of viral load/immune marker, we fit a model by cross validation. We chose the best fit model from the five classes, but only the two simplest models were ever selected (82/96=85% were linear, and 14/96=15% were exponential). We list the 96 models sorted by adjusted R-squared value (Table S5). The distribution of the adjusted R-squared $\left(R_{a}^{2}\right)$ values are: 28/96=29% have $0.4\leq R_{a}^{2}<0.53,;49/96=51\%$ have $0.2\leq R_{a}^{2}<0.4$; and 19/96 have $0.1\leq R_{a}^{2}<0.2$. Of the models with $R_{a}^{2}\geq 0.4$, most used AUC (20/28=71%) and most used nasal swabs (24/28=86%), but they had similar amounts using either type of RT-qPCR assay (15 usedN1 and 13 used Subgenomic E). There was no immune marker that had more than 4/28=14%. In Figure 11, we plot the best fitting model (exponential on [log10 MN| Day 0] by [AUC| BAL| N1]) and another representative model (the seventh best fitting model, linear on [log10 PsVNA ID80| Day -14] by [AUC |Nasal Swab |Subgenomic E]). The exponential model in Figure 11 estimates that a 10-fold increase in its immune marker decreases the AUC viral load measure by 18% (fold-change = 0.82 [95% CI: 0.79, 0.84]), while the linear model in Figure 11 estimates that a 10-fold increase in that immune marker decreases the AUC viral load measure by 4 (change in AUC = −4.0 [95% CI: −4.73, −3.26]). Figure 11 and Table S5 show that either the exponential or linear model can be a good fit, and there is not one sampling location (Nasal, OP, or BAL), viral assay (N1 or Subgenomic E), immune assay, or immune marker day (Day -14 or Day 0) that is substantially better than another for estimating the relationship between immune measure and viral load.

### Percent of Variance Explained

In the previous section we used simple models that only used one immune marker to explain one of the viral load measurements. Here, we examined whether information about the type of vaccine and its dose can appreciably improve the model fit, and found that the simple models without terms for vaccine type or dose fit almost as well as the more complicated models.

**Figure 12.**
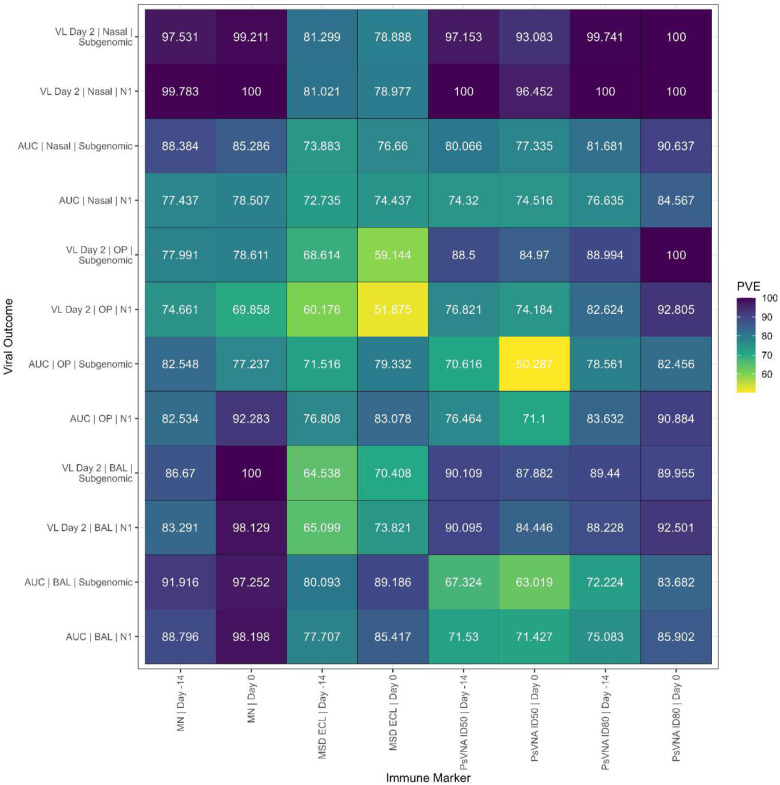
Percent of Variance Explained. Percent of Variance Explained (i.e., 100 times the ratio of adjusted R-squared values) comparing simple models to ones allowing different linear predictor effects for each vaccine type. Values greater than 100% are set to 100%.

We examined this issue using the percent of variance explained (PVE) by the simple models, using the previously chosen model class, compared to the associated models that allow different linear predictor effects for each vaccine type. We plotted the PVE values in Figure 12 (95% confidence intervals in Table S6) for the 96 viral load/immune marker combinations. The PVE estimates ranged from 50.29% to 100% with most (54/96) viral load/immune marker combinations giving PVE above 80%. For the two models of Figure 11 the associated PVE were 98.2% (95% CI: 96.63%, 100%) for the left panel and 81.7% (95% CI: 74.5%, 92.9%) for the right panel.

### Cross Validation Prediction using Vaccine Product

Previously, we used cross validation to choose among five model classes for each of the 96 viral load/immune marker pairs. In this section, we assume that the previously chosen model class is correct, and then re-estimate the model parameters leaving out all data from one of the vaccine products. Using the estimated model, we predict the viral load for animals that received the vaccine product not included in the model fit. We then calculate the random marginal agreement coefficient (RMAC) concordance correlation (CC_RMAC_) to see how well the predicted viral load agrees with the observed viral load. In general, we observed positive agreement, but it was not always significant. The results are in Tables S7–S10.

Consider the CC_RMAC_ values associated with the best fit model depicted in the left panel of Figure 11 (AUC | BAL | N1/log10 MN | Day 0). Leaving out each of the vaccine products in turn, we get CC_RMAC_ values for Sanofi (CC_RMAC_ = 0.59, 95% CI 0.30, 0.78), Moderna (CC_RMAC_ = 0.44, 95% CI 0.07, 0.70), Janssen (CC_RMAC_ = 0.46, 95% CI 0.10, 0.72), and Novavax (CC_RMAC_ = 0.25, 95% CI −0.25, 0.49). The estimated agreement for all four vaccine products was positive, although the CC_RMAC_ for Novavax was not significantly different from 0. For the right panel of Figure 11 (AUC | Nasal | subgenomic/log10 PsVNA ID80 | Day -14) we get CC_RMAC_ values for Sanofi (CC_RMAC_ = 0.35, 95% CI 0.04, 0.63), Moderna (CC_RMAC_ = 0.40, 95% CI 0.12, 0.57), Janssen (CC_RMAC_ = 0.04, 95% CI −0.29, 0.34), and Novavax (CC_RMAC_ = 0.35, 95% CI −0.47, 0.64); only Sanofi and Moderna were significantly different from 0.

**Figure 13.**
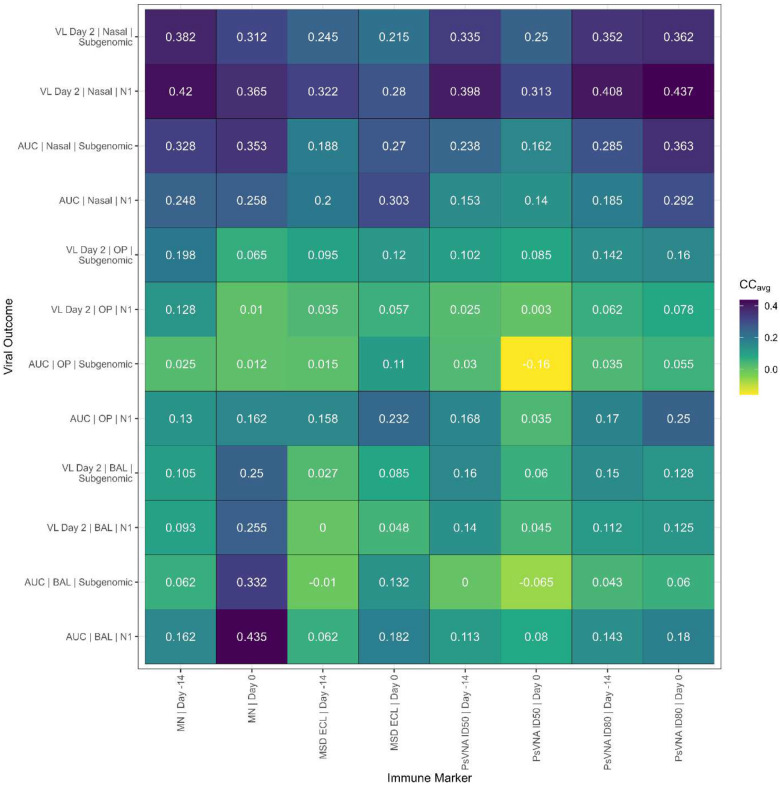
Average concordance correlation. Average random marginal agreement coefficient (RMAC) concordance correlation (CC_RMAC_) for each of the 96 immune marker and viral outcome combinations. CC_RMAC_ is calculated by re-estimating the model parameters leaving out all data from one of the vaccine products, then seeing how well the predicted viral load for animals that received the vaccine product not included in the model fit agrees with the observed viral load. The four CC_RMAC_ for each immune marker/viral outcome combination are averaged and shown above.

We summarize the separate tables for the four vaccines (Tables S7–S10) by averaging over each element to get the average RMAC concordance correlations shown in Figure 13. Nearly all 96 viral load/immune marker pairs have positive agreement (average CC_RMAC_>0).

### Vaccine Efficacy by Viral Load

**Figure 14.**
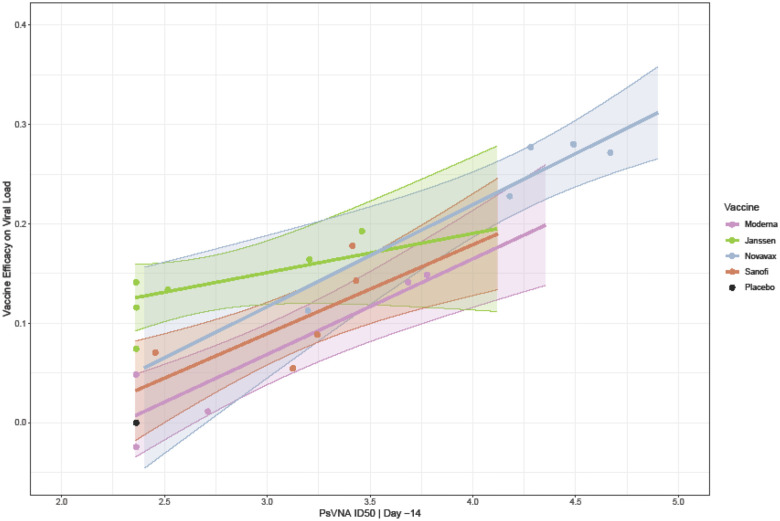
Vaccine efficacy comparing viral load by AUC | Nasal |Subgenomic to PsVNA ID50 at Day – 14. Each dot represents the mean immune marker and mean viral load for one dose group in one type of vaccine. The lines represent the models, and the shaded regions represent the 95% confidence intervals, with the x-axis range matching the range of the individual PsVNA ID50 |Day -14 values. The black dot represents the placebo arm, which has all animals below the limit of detection for the immune marker and the ratio is 1 by definition, so the VEVL is 0. For Janssen, we combined the one-dose and two-dose groups.

**Figure 15.**
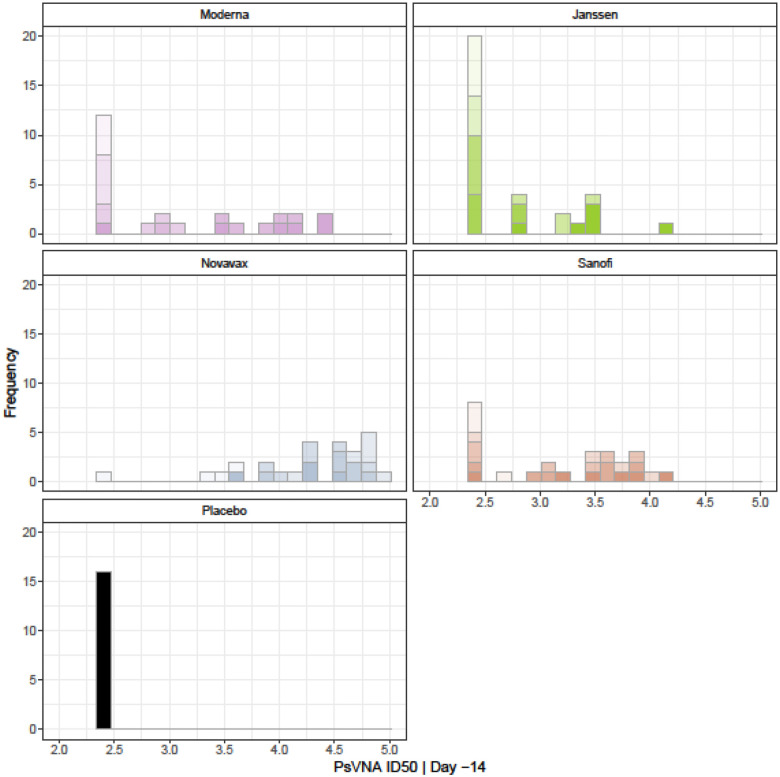
Histograms for PsVNA ID50| Day –14 for the different vaccine types. Different shades denote different dose groups with darker colors representing higher doses (based on the s=½ dose score for Janssen).

Many of the previous analyses estimate associations between immune markers and viral load outcomes (CoR analyses), but here we explicitly model the relationship between immune markers and vaccine efficacy (CoP analysis). We ran an analysis to mimic the analysis presented in the figure from Gilbert, et al, 2022 [53]. That paper modeled vaccine efficacy based on time to COVID-19 from several human trials using different vaccines using neutralizing antibody titer (IU50/ml). Figure 14 shows the vaccine efficacy by viral load, with the immune marker measured using PsVNA ID50 | Day −14 and expected viral load using AUC | Nasal Swab | subgenomic. The VEVL modeled for all vaccines showed positive slopes, and all except Janssen have confidence regions that exclude the zero slope, showing a significant effect of the immune marker. Figure 15 gives histograms of the PsVNA ID50 | Day –14.

## Discussion

Unique to this study is that four different vaccines were tested in a single GLP, randomized study; therefore, we controlled for many variables (animal population, facility, assays, timing). Individual animal data were combined to create a series of viral load prediction models, and each model was applied across the four different vaccines, providing for a larger sample size than with a single vaccine NHP study. There were 252 immune marker samples (2 time points for each of n=126 animals) measured on 4 assays, showing a clear strong correlation among immune markers (Figure 6). The large sample size gave prediction models with tight confidence intervals (see e.g., Figure 11). Further, the percent variance explained values of 80% or larger showed that the immune markers (when they are one of 28 out of 96 better fitting models) accounted for nearly all the variance that can be explained even if we added different dose effects for the different vaccines (Figure 12).

The four vaccines represent different platforms (mRNA, replication-deficient human adenovirus, or recombinant protein) which is a strength of the study, since it can posit immune markers that can act across different platforms. However, the different platforms pose challenges. First, it is difficult to compare dose schedules and doses between different vaccines, because of the different platforms. This is observed in the different spread of the range of doses across the four vaccines (in addition to the single dose groups for the Janssen vaccine). The different strength of dose responses also made comparisons between vaccines difficult. For example, in Figure 8 it appears that the doses chosen for Moderna give increasing levels of immune markers, while the doses for the other vaccines do not show as strong a dose response for the immune markers. These dose choices could be the reason that in Table S4 the Moderna dose effect is non-significant [p=0.114], compared to highly significant dose effects for the other three vaccines [p=0.002]. Another issue with the dose scores is that the increase from placebo dose score (0) to the minimum dose for each vaccine (1), is the same as any 10-fold increase in dose regardless of whether the lowest dose is low or high relative to humanized or protective dose level (see Supplemental Figure S5).

Using the data from three vaccines to build a model to predict the viral load response in animals that received the other vaccine produced positive agreement coefficients; however, several of them were not significantly different from 0. This could be an analysis that is overly ambitious, since it requires not only that the same immune marker predicts well for all 4 vaccine products but is also hindered by assay measurement error and large within-group variability in the viral load measurements.

A strength of this study is that the statistical analysis plan was pre-specified, and all the mandatory analyses are available in the supplement (SAP report[52]). Motivations for extra analyses are explicitly referenced in this paper. Nevertheless, many of the analyses that were pre-specified did not provide much additional insight and are not presented in this paper. One example was the way the one and two-dose groups are handled for the Janssen vaccine. We only presented the method where we modify the one dose by halving the doses before calculating the dose scores. Two alternative analyses were performed: (1) all Janssen doses were set to the dose delivered at a single timepoint (effectively ignoring the fact that some animals were given only one dose), or (2) animals that received one dose were removed from the analysis. In general, the three ways of handling the Janssen dosages produced analyses that were very similar. Another example of analyses that are not presented here are analyses where the viral load measurements are categorized into binary responses (detectable or not). These binary response model analyses were generally not helpful because nearly all the animals and viral load measurements had detectable virus measurements.

The analyses presented here combined with the clinical studies literature [53–57] provide strong support for microneutralization measured on the day of challenge (MN Day 0) as a univariate correlate of protection, when protection is defined as reduction in the viral load measured in the lower respiratory tract (BAL). This is supported by this immune marker having the highest PVE for all four of the BAL viral load models (Day 2 or AUC crossed with Subgenomic E or N1). In addition, MN at Day 0 resulted in the highest concordance measures for the four models in the leave-one-vaccine-out cross-validation analysis.

The results are not as clear for viral load measured in upper respiratory tract (nasal swab analyses). Although, the pseudovirus neutralization (PsVNA) ID_80_ measured on the day of challenge had the highest PVE for all four models, MSD ECL and MN Day-14 sometimes had the highest concordance in the leave-one-vaccine-out cross-validation analysis. This may be due to viral load measurements from Nasal samples on Day 2 being influenced by the intranasal component of the challenge.

Previous work [53] proposed that serum anti-spike IgG concentration and anti-SARS-CoV-2 neutralizing antibody titers as CoPs for vaccines against symptomatic COVID-19. In this study there is no measure of symptomatic COVID-19 that could be used to define protection and instead, the possibly more informative, reduction in the continuous qRT-PCR viral load was used to define protection. Also, of note, the immune measure PsVNA ID50 that was the focus of the previous work was somewhat hindered in this NHP study by a relatively high LOD for the NHP assay.

The analyses presented here show that these four assays (live virus microneutralization assay, Meso scale discovery electrochemiluminescence immunoassay, and pseudovirus neutralizing assay using 50% or 80% inhibitory dose) each at two timepoints were highly correlated. In addition, the PVE analysis shows that many other immune measures have PVE estimates that were not significantly less than those for the immune measures mentioned above. Thus, the study provides evidence for all four of these immune measures as correlates of protection.

## Supplementary Material

1

Supplement: Supplementary figures, tables, statistical method details, and genetic sequence details. The statistical analysis plan (SAP), and SAP report are available at [52].

## Figures and Tables

**Figure 3.**
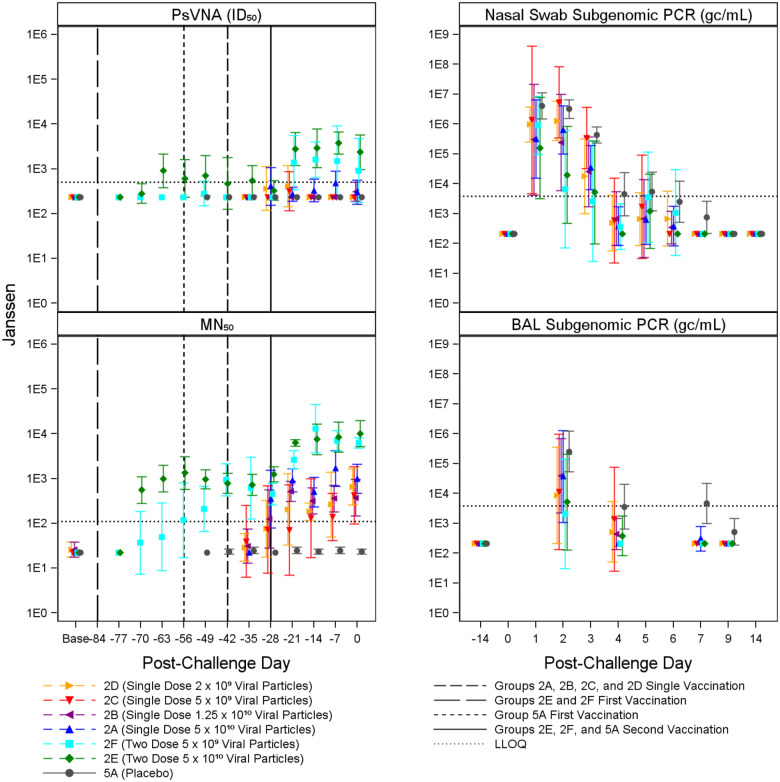
Summary of Janssen PsVNA_50_, MN_50_, and Subgenomic PCR Results. Geometric mean and associated 95% confidence intervals for PsVNA (ID_50_) and MN_50_ immune markers and BAL and nasal swab Subgenomic E PCR viral load, shown by dose and post-challenge day for Janssen and placebo (grey). Both the single and two dose groups are displayed. Dose groups are ordered by lowest (2D, yellow) to highest (2A, blue) for the single dose group, and lowest (2F, light blue) to highest (2E, green) for the two-dose group. The dotted horizontal line indicates LLOQ. Vaccination schedules for the different groups are shown by the vertical lines.

**Figure 4.**
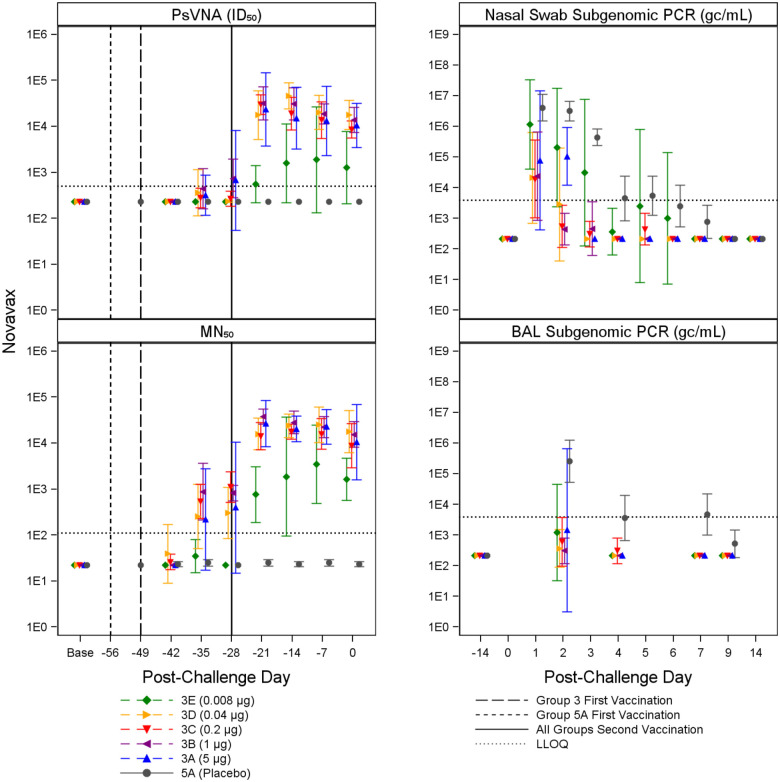
Summary of Novavax PsVNA_50_, MN_50_, and Subgenomic PCR Results Geometric mean and associated 95% confidence intervals for PsVNA (ID_50_) and MN_50_ immune markers and BAL and nasal swab Subgenomic E PCR viral load, shown by dose and post-challenge day for Novavax and placebo (grey). Dose groups are ordered by lowest (3E, green) to highest (3A, blue). The dotted horizontal line indicates LLOQ. Vaccination schedules for the different groups are shown by the vertical lines.

**Figure 5.**
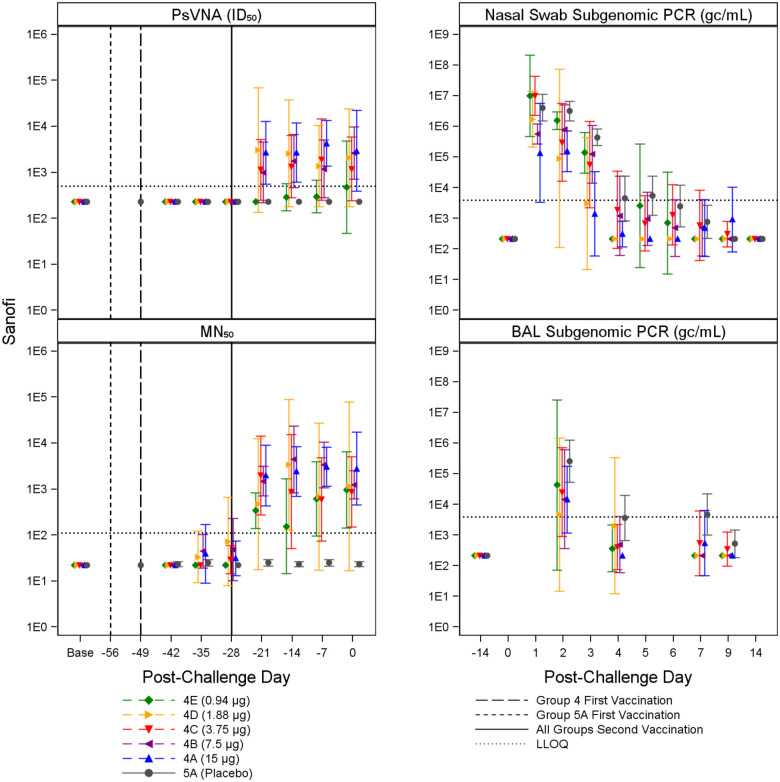
Summary of Sanofi PsVNA_50_, MN_50_, and Subgenomic PCR Results. Geometric mean and associated 95% confidence intervals for PsVNA (ID_50_) and MN_50_ immune markers and BAL and nasal swab Subgenomic E PCR viral load, shown by dose and post-challenge day for Sanofi and placebo (grey). Dose groups are ordered by lowest (4E, green) to highest (4A, blue). The dotted horizontal line indicates LLOQ. Vaccination schedules for the different groups are shown by the vertical lines.
