# Supplementary material for: Humoral Immune Correlates Analysis of Four Vaccines Against SARS-CoV-2 in Rhesus Macaques
Source: Res Sq. 2026 Apr 30:rs.3.rs-9404931. Preprint. [Version 1] doi: 10.21203/rs.3.rs-9404931/v1 (PMC13142646; doi:10.21203/rs.3.rs-9404931/v1)
Supplement: 1 [file NIHPPRS9404931V1-supplement-1.pdf]

Supplement to: **Humoral Immune Correlates Analysis of Four Vaccines Against SARS-CoV-2 in Rhesus Macaques**

Michael P. Fay[1], Allyson Mateja[2], Chris Cirimotich[3], Jennifer Garver[3], Ying-Liang Chou[3], Wantong Du[3], Michael Anderson[3], April Brys[4], Carol Sabourin[5], James Long[5], James Little[4], Sara Duncan[4], Jason Mott[5], Tanima Sinha[4], Nina Malkovich[4], Greg V. Stark[5], Daniel C. Sanford[3]

[1] NIH Biostatistics Research Branch/DCR/NIAID, Rockville, MD, USA.

[2] Clinical Monitoring Research Program Directorate, Frederick National Laboratory for Cancer Research, Frederick, MD, USA.

[3] Battelle Memorial Institute, Columbus OH, USA.

[4] Biomedical Advanced Research and Development Authority (BARDA), Washington, DC, USA.

[5] Tunnell Government Services, Inc, Supporting Biomedical Advanced Research and Development Authority (BARDA), Washington, DC, USA.

September 17, 2025

Record locator#UID-2026-03-13-630

Supplemental Figures

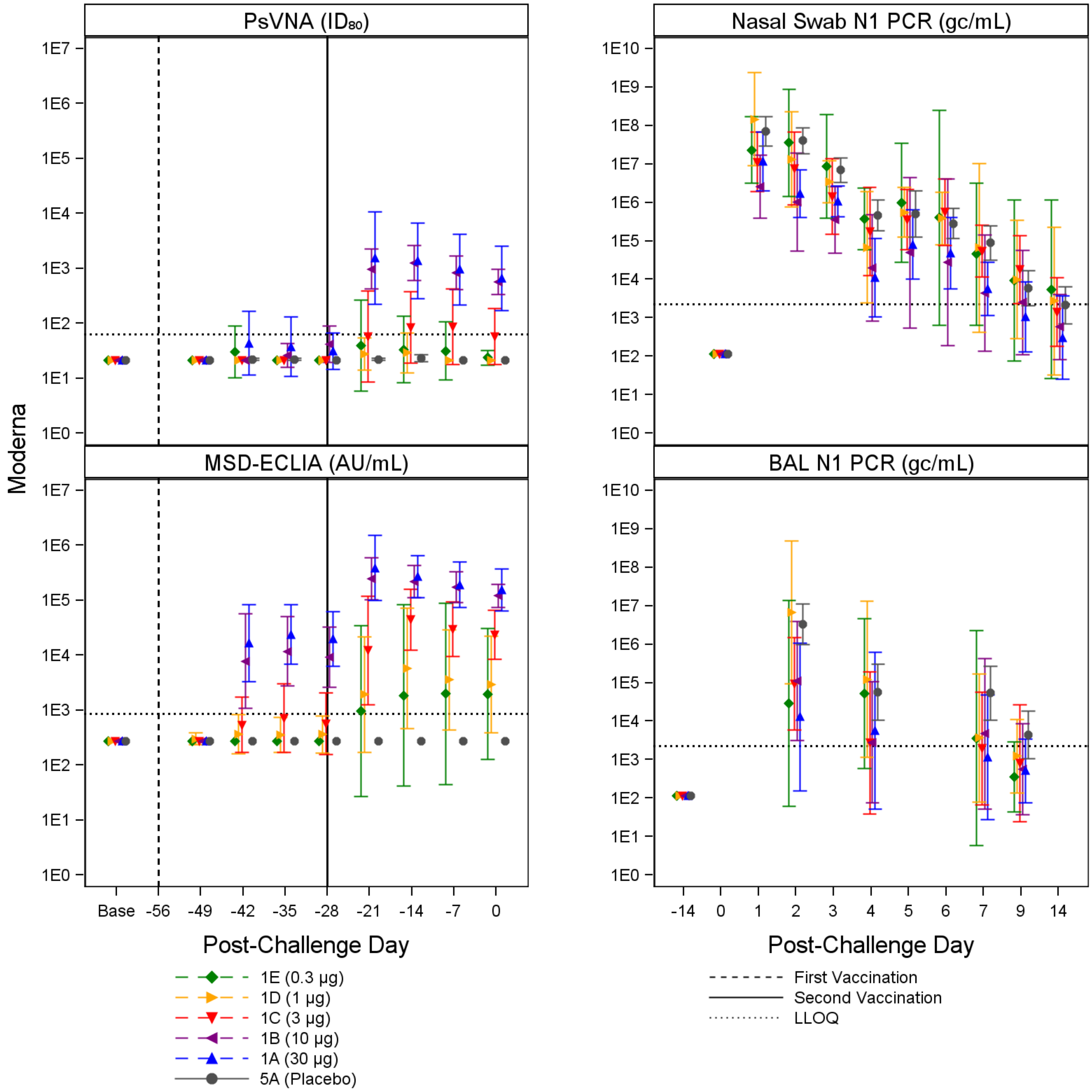

Supplemental Figure 1. Summary of Moderna PsVNA$_{80}$, MSD-ECLIA, and N1 PCR Results. Geometric mean and associated 95% confidence intervals for PsVNA (ID$_{80}$) and MSD-ECLIA immune markers and BAL and nasal swab N1 PCR viral load, shown by dose and post-challenge day for Moderna and placebo (grey). Dose groups are ordered by lowest (1E, green) to highest (1A, blue). The dotted horizontal line indicates LLOQ. The first vaccination is shown with a dashed vertical line, and the second vaccination is shown with a solid vertical line.

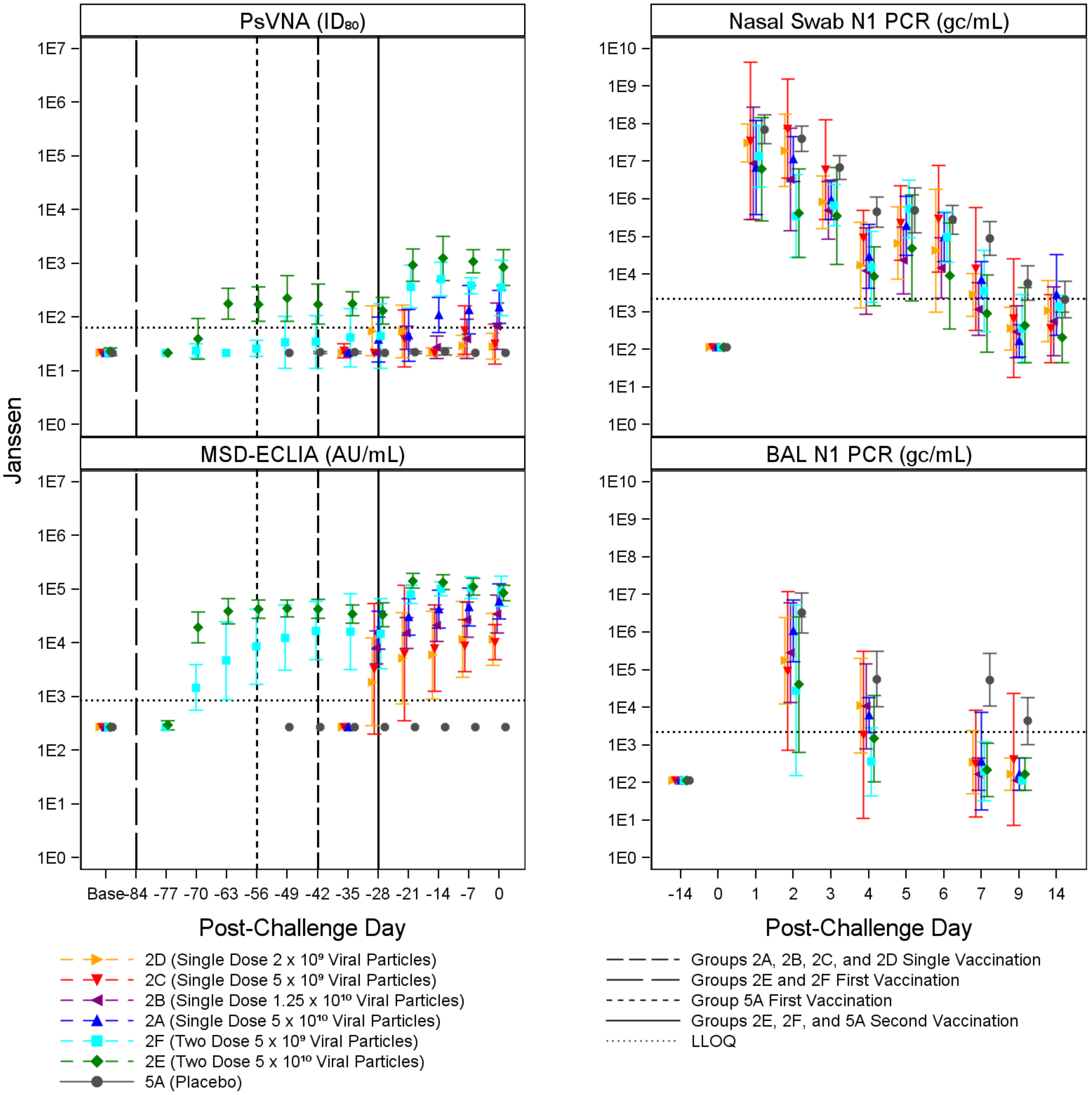

Supplemental Figure 2. Summary of Janssen PsVNA$_{80}$, MSD-ECLIA, and N1 PCR Results. Geometric mean and associated 95% confidence intervals for PsVNA (ID$_{80}$) and MSD-ECLIA immune markers and BAL and nasal swab N1 PCR viral load, shown by dose and post-challenge day for Janssen and placebo (grey). Both the single and two dose groups are displayed. Dose groups are ordered by lowest (2D, yellow) to highest (2A, blue) for the single dose group, and lowest (2F, light blue) to highest (2E, green) for the two-dose group. The dotted horizontal line indicates LLOQ. Vaccination schedules for the different groups are shown by the vertical lines.

Record locator#UID-2026-03-13-630

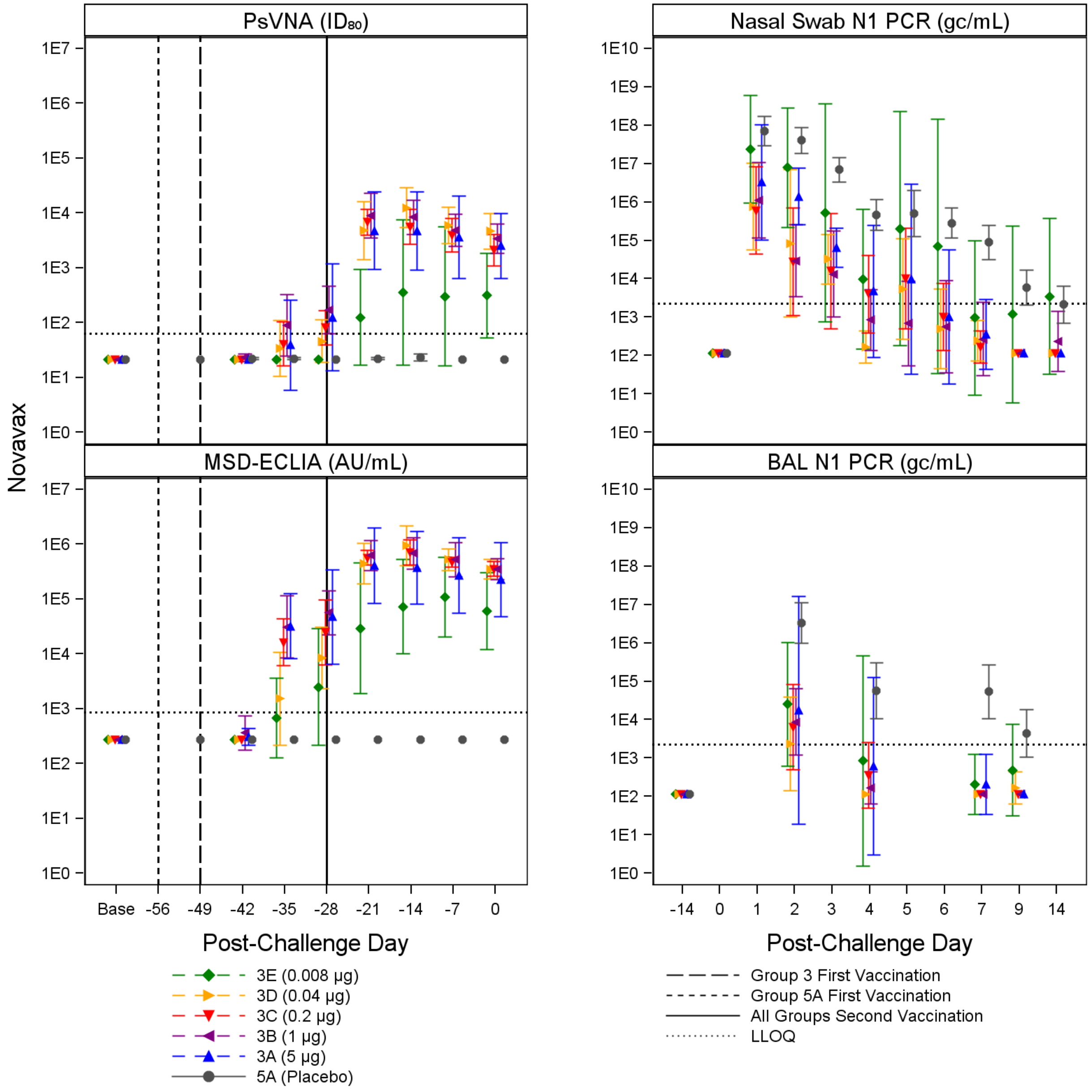

Supplemental Figure 3. Summary of Novavax PsVNA$_{80}$, MSD-ECLIA, and N1 PCR Results. Geometric mean and associated 95% confidence intervals for PsVNA (ID$_{80}$) and MSD-ECLIA immune markers and BAL and nasal swab N1 PCR viral load, shown by dose and post-challenge day for Novavax and placebo (grey). Dose groups are ordered by lowest (3E, green) to highest (3A, blue). The dotted horizontal line indicates LLOQ. Vaccination schedules for the different groups are shown by the vertical lines.

Record locator#UID-2026-03-13-630

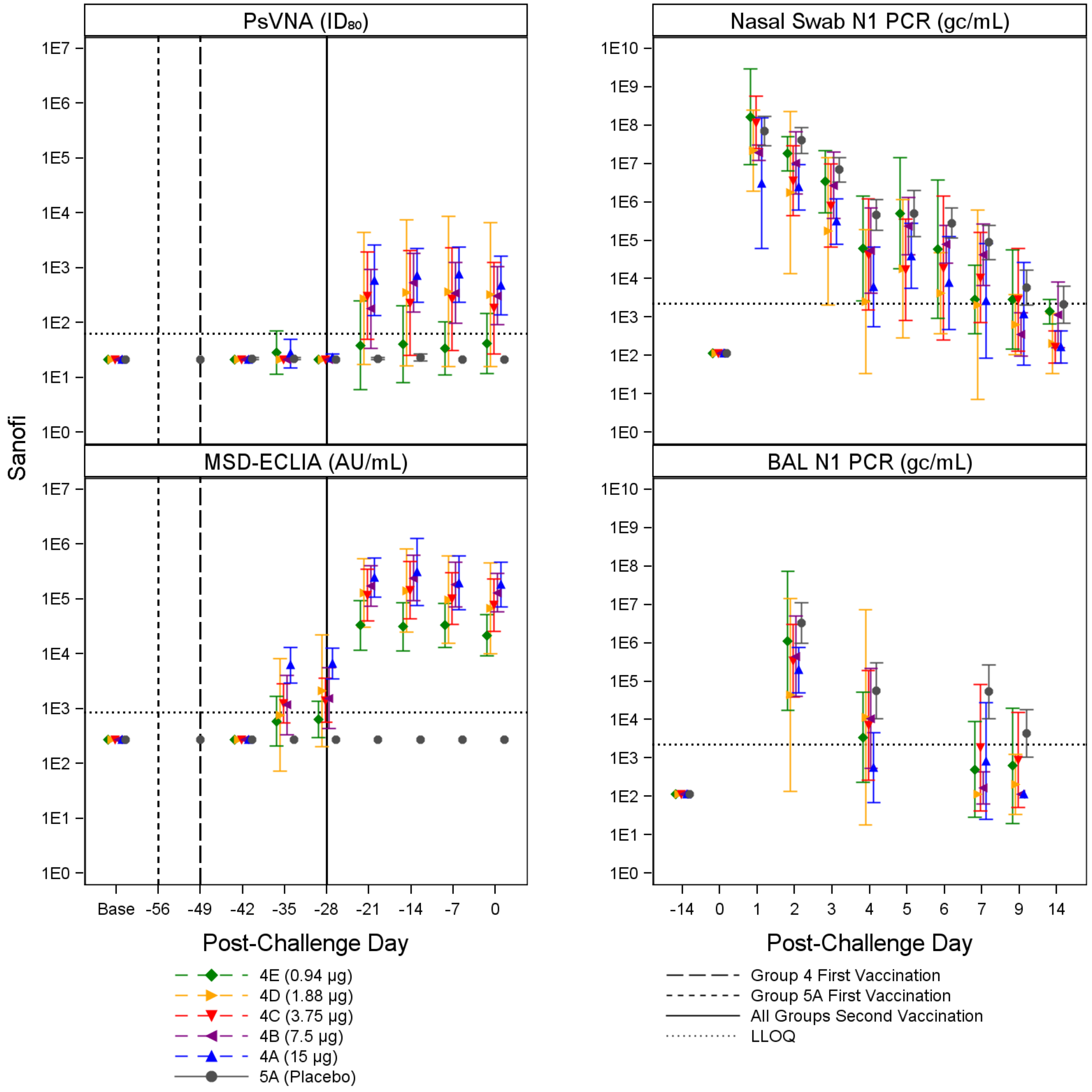

Supplemental Figure 4. Summary of Sanofi PsVNA$_{80}$, MSD-ECLIA, and N1 PCR Results. Geometric mean and associated 95% confidence intervals for PsVNA (ID$_{80}$) and MSD-ECLIA immune markers and BAL and nasal swab N1 PCR viral load, shown by dose and post-challenge day for Sanofi and placebo (grey). Dose groups are ordered by lowest (4E, green) to highest (4A, blue). The dotted horizontal line indicates LLOQ. Vaccination schedules for the different groups are shown by the vertical lines.

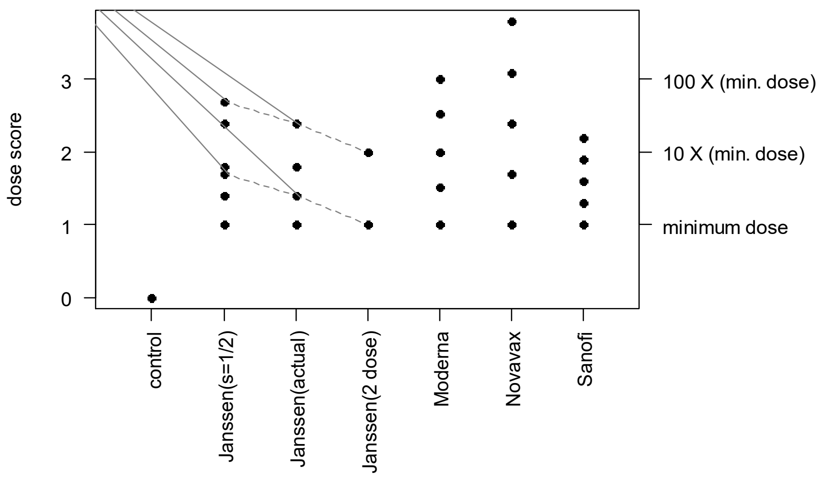

Supplemental Figure 5: Dose scores for each vaccine. Dose score for control is 0 and dose score for dose D is scored as $\log_{10}\left(\frac{10*D}{D_{min}}\right)$, where D_{min} is the minimum dose among all dosages for that vaccine. So, dose score of 1 is the minimum dose, and dose score of 2 is 10 times the minimum dose within a vaccine. The Janssen vaccine is scored 3 different ways: Janssen (s=1/2) had each single immunization dosage group have its dosage halved before scoring, Janssen (actual) uses the dose for each immunization regardless if it was a two dose or single dose group, and Janssen (2 dose) only includes the two dose immunization groups. Only Janssen (s=1/2) is discussed in the main paper.

Record locator#UID-2026-03-13-630

Supplemental Statistical Methods

Extra Analyses using Percent Variance Explained

As an appendix to SAP section 6.5, we repeated the Percent Variance Explained (PVE) analysis using binned dose scores. Previously, we were describing the association between the virus-specific antibody and the viral load with two models: the base model and the base+dose model. The base model predicts the viral load using the virus-specific antibody. The base+dose model is a linear model that allows a separate line of prediction of viral load by virus-specific antibody for each dose score (s=1/2). As an appendix, the dose scores (s=1/2) are binned as follows: 0, 1, (1, 1.5], (1.5, 2], (2, 2.5], (2.5, 3], > 3. This is being done to attempt to improve the performance of the bootstrap confidence intervals, which do not always include the full estimate. In the base+dose model, information regarding the identity of the vaccine administered is not included in the analysis, except in information captured in the dose score. For the second model, termed the base+dose+vacc model, separate effects for each specific vaccine/dose score combination (i.e., a different parameter for each specific vaccine/dose) will be included. This analysis was also repeated using larger binned dose scores (s=1/2): 0, 1, (1, 2], >2. This was done to increase the amount of data in each bin, after splitting by vaccine. As an additional analysis, we compare the base model to the base+dose+vacc model. Details of the base model are explained previously. The base+dose+vacc model used here is slightly different than previously described. In this model, the dose score (s=1/2) is used as a numeric value, with a separate effect for each vaccine. Linear models for numeric viral load (more complicated models, regardless of what was selected by the cross-validation procedure, are not used) will be employed. The percentage of variance (PVE) is 100 times the adjusted R squared from the base model over the adjusted R squared values from the base+dose+vacc model. Confidence intervals on the PVE are calculated by using bias-adjusted nonparametric bootstrap methods (using 5000 replicates).

Details on Simulations to Justify Cross-validation Methods

A dataset was created using means and variances approximated from Mercado, et al (2020) Figure 6a (week 4), with some variability added between manufacturers' products.

1000 datasets were simulated from each of the 6 model types (linear, exponential, splines with 5 degrees of freedom, splines with 10 degrees of freedom, 4-parameter logistic, and segmented). The simulated datasets were created by fitting our dataset to each model, then adding random noise to the predicted values. For each simulated dataset, 1000 replicates of cross-validation (CV) were performed. The cross-validation is done by splitting the dataset into a training (90%) and test (10%) set. Each of the 6 model types are fit to the training set, then the fitted model is applied to the test set, and the R-squared is calculated. From each repeated CV, we determine which model has the highest R-squared. We can then determine, out of the 1000 CVs, which model was selected the most. We choose this model as the best fit to the data.

After simulating 1000 datasets from a linear model and doing the repeated CV for each of those datasets, we found that the linear model was only selected as the best fit for 58.3% of the datasets. The model of splines with 10 degrees of freedom was selected for 21.1% of the datasets, as it is more flexible. When we switch to using adjusted R-squared, the linear model was selected for 99.7% of the datasets. Similar for the exponential model, splines with 5 degrees of freedom, and 4-parameter logistic models, the correct model was selected for all 1000 datasets; for the segmented model, the correct model was selected for 97.2% of the datasets. Therefore, we switched to using adjusted R-squared to evaluate the cross-validation.

When evaluating the model using splines with 10 degrees of freedom, the correct model was selected for only 5.6% of the datasets when using adjusted R-squared. The model using splines with 5 degrees of season was instead selected in 94.4% of the datasets. As the adjusted R-squared worked well for all other models, we removed the model using splines with 10 degrees of freedom from list of models from which we can select.

In addition, for each of the 1000 simulated datasets, we use the selected model to get 95% confidence intervals around predicted values (at x = 2, 2.5, 3, 3.5, 4, 4.5) that represent the spread of the correlates data. At each of the 6 points, we record if the true predicted value is inside the confidence interval. Coverage was good (approximately 95%) at each of the 6 points for the linear, exponential, and 4-parameter logistic models, as well as the model using splines with 5 degrees of freedom. Coverage was lower than 95% using the segmented model, but improved to approximately 95% when pointwise confidence intervals were calculated using nonparametric bootstrap, rather than the built-in predict function in R. Therefore, if the segmented model is selected during the cross-validation procedure, we will use nonparametric bootstrap to calculate the confidence intervals.

We repeated the simulation procedure for the logistic models: logistic regression, splines with 5 degrees of freedom, and segmented regression. After simulating 1000 datasets from a logistic model and using the adjusted coefficient of discrimination to select the best model from the cross-validation procedure, the correct model was selected only 85% of the time, although the coverage was 95% at the 6 predicted values (at x = 2, 2.5, 3, 3.5, 4, 4.5). The model of splines with 5 degrees of freedom was correctly selected only 20.7% of the time, although coverage of the predicted values was still high. When using segmented regression, the correct model was selected only 10.3% of the time. Coverage was less than 95% at all 6 points, especially at the first point of x=2 (coverage was only 13.9%). We proceeded to simulate 1000 datasets from a segmented regression model, but skipping the cross-validation step, and getting the coverage at the 6 predicted values, assuming we know the true model was segmented. When using the built-in predict function in R to get the confidence intervals, coverage was still less than 95%. Using nonparametric bootstrap to get the confidence intervals vastly improved the coverage; 95% coverage was achieved at 5 points, x = 2.5, 3, 3.5, 4, 4.5. At the first point, x=2, coverage was only 82.6%, which was low but a large improvement over the other methods. We hypothesize that this is due to the estimated breakpoints in the model being close to x=2. Therefore, when the segmented model is selected during cross-validation, we will obtain the confidence intervals around the predicted values using nonparametric bootstrap.

Supplemental Genetic Sequence Information

Synthetic RNA used in RT-qPCR Nucleocapsid Protein (N1) Genomic Analysis:

(5’UGUCUGAUAAUGGACCCCAAAAUCAGCGAAAUGCACCCCGCAUUACGUUUGGUGGACCCUCAGAUUCAAC
UGGCAGUAACCAGAAUGGAGAACGCAGUGGGGCGCGAUCAAAACAACGUCGGCCCCAAGGUUUACCCAAUAA
UACUGCGUCUUGGUUCACCGCUCUCACUCAACAUGGCAAGGAAGACCUUAAAUUCCCUCGAGGACAAGGCG
UUCCAAUUAACACCAAUAGCAGUCCAGAUGACCAAAUUGGCUACUACCGAAGAGCUACCAGACGAAUUCGUG
GUGGUGACGGUAAAAUGAAAGAUCUCAGUCCAAGAUGGUAUUUCUACUACCUAGGAACUGGGCCAGAAGC
UGGACUUCCCUAUGGUGCUAACAAAGACGGCAUCAUAUGGGUUGCAACUGAGGGAGCCUUGAAUACACCAA
AAGAUCACAUUGGCACCCGCAAUCCUGCUAACAAUGCUGCAAUCGUGCUACAACUUCCUCAAGGAACAACAU
UGCCAAAAGGCUUCUACGCAGAAGGGAGCAGAGGCGGCAGUCAAGCCUCUUCUCGUUCCUCAUCACGUAGU
CGCAACAGUUCAAGAAAUUCAACUCCAGGCAGCAGUAGGGGAACUUCUCCUGCUAGAAUGGCUGGCAAUGG
CGGUGAUGCUGCUCUUGCUUUGCUGCUGCUUGACAGAUUGAACCAGCUUGAGAGCAAAAUGUCUGGUAAA
GGCCAACAACAACAAGGCCAAACUGUCACUAAGAAAUCUGCUGCUGAGGCUUCUAAGAAGCCUCGGCAAAAA
CGUACUGCCACUAAAGCAUACAAUGUAACACAAGCUUUCGGCAGACGUGGUCCAGAACAAACCCAAGGAAAU
UUUGGGGACCAGGAACUAAUCAGACAAGGAACUGAUUACAAACAUUGGCCGCAAAUUGCACAAUUUGCCCC
CAGCGCUUCAGCGUUCUUCGGAAUGUCGCGCAUUGGCAUGGAA 3’)

Synthetic RNA used in RT-qPCR Envelope Protein (E) Subgenomic Analysis:

(5’GGGGGGGCGAUCUCUUGUAGAUCUGUUCUCUAAACGAACUUAUGUACUCAUUCGUUUCGGAAGAGACA
GGUACGUUAAUAGUUAAUAGCGUACUUCUUUUUCUUGCUUUCGUGGUAUUCUUGCUAGUUACACUAGCCA
UCCUUACUGCGCUUCGAUUGUGUGCGUACUGCUGCAAUAUUGUUAACGUGAGUCUUGUAAAACCUUCUUU
UUACGUUUACUCUCGUGUUAAAAAUCUGAAUUCUUCUAGAGUUCCUGAUCUUCUGGUCUAAACGAACUAA
AUAUUAUAUUAGUUUUUCUGUUUGGAACUUUAAUUUUAGCCAUGGCAGA3’)

Record locator#UID-2026-03-13-630

Supplemental Tables

Table S1. Spearman correlations (with 95% confidence intervals) of virus-specific antibodies.

	MN | Day -14	MN | Day 0	MSD ECL | Day -14	MSD ECL | Day 0	PsVNA ID50 | Day -14	PsVNA ID50 | Day 0	PsVNA ID80 | Day -14	PsVNA ID80 | Day 0
MN | Day -14	-	-	-	-	-	-	-	-
MN | Day 0	0.89 (0.85, 0.92)	-	-	-	-	-	-	-
MSD ECL | Day -14	0.93 (0.9, 0.95)	0.89 (0.84, 0.92)	-	-	-	-	-	-
MSD ECL | Day 0	0.91 (0.88, 0.94)	0.89 (0.84, 0.92)	0.98 (0.97, 0.98)	-	-	-	-	-
PsVNA ID50 | Day -14	0.89 (0.85, 0.92)	0.86 (0.8, 0.9)	0.91 (0.88, 0.94)	0.89 (0.84, 0.92)	-	-	-	-
PsVNA ID50 | Day 0	0.88 (0.83, 0.91)	0.86 (0.81, 0.9)	0.9 (0.86, 0.93)	0.89 (0.85, 0.92)	0.93 (0.9, 0.95)	-	-	-
PsVNA ID80 | Day -14	0.92 (0.89, 0.94)	0.87 (0.83, 0.91)	0.93 (0.9, 0.95)	0.91 (0.88, 0.94)	0.96 (0.94, 0.97)	0.92 (0.89, 0.94)	-	-
PsVNA ID80 | Day 0	0.93 (0.9, 0.95)	0.91 (0.88, 0.94)	0.93 (0.9, 0.95)	0.94 (0.91, 0.96)	0.93 (0.91, 0.95)	0.94 (0.92, 0.96)	0.95 (0.92, 0.96)	-

Table S2. Spearman correlations (with 95% confidence intervals) of viral load outcomes.

	AUC | BAL | N1	AUC | BAL | Subge-nomic	AUC | Nasal | N1	AUC | Nasal | Subge-nomic	AUC | OP | N1	AUC | OP | Subge-nomic	VL Day 2 | BAL | N1	VL Day 2 | BAL | Subge-nomic	VL Day 2 | OP | N1	VL Day 2 OP | Subge-nomic	VL Day 2 | Nasal | N1	VL Day 2 | Nasal | Subge-nomic
AUC | BAL | N1	-	-	-	-	-	-	-	-	-	-	-	-
AUC | BAL | Subgenomic	0.88 (0.83, 0.91)	-	-	-	-	-	-	-	-	-	-	-
AUC | Nasal | N1	0.61 (0.49, 0.71)	0.59 (0.46, 0.69)	-	-	-	-	-	-	-	-	-	-
AUC | Nasal | Subgenomic	0.62 (0.5, 0.72)	0.55 (0.42, 0.66)	0.87 (0.82, 0.91)	-	-	-	-	-	-	-	-	-
AUC | OP | N1	0.73 (0.64, 0.81)	0.61 (0.49, 0.71)	0.78 (0.7, 0.84)	0.76 (0.67, 0.82)	-	-	-	-	-	-	-	-
AUC | OP | Subgenomic	0.7 (0.6, 0.78)	0.59 (0.46, 0.69)	0.69 (0.59, 0.78)	0.69 (0.59, 0.77)	0.89 (0.85, 0.92)	-	-	-	-	-	-	-
VL Day 2 | BAL | N1	0.86 (0.81, 0.9)	0.88 (0.83, 0.91)	0.48 (0.33, 0.61)	0.47 (0.32, 0.6)	0.57 (0.44, 0.68)	0.55 (0.41, 0.66)	-	-	-	-	-	-
VL Day 2 | BAL | Subgenomic	0.82 (0.75, 0.87)	0.93 (0.9, 0.95)	0.48 (0.33, 0.6)	0.47 (0.32, 0.6)	0.55 (0.42, 0.66)	0.52 (0.37, 0.63)	0.93 (0.9, 0.95)	-	-	-	-	-
VL Day 2 | OP | N1	0.43 (0.27, 0.56)	0.35 (0.19, 0.5)	0.45 (0.3, 0.58)	0.47 (0.32, 0.59)	0.52 (0.38, 0.64)	0.57 (0.44, 0.68)	0.31 (0.14, 0.46)	0.27 (0.1, 0.42)	-	-	-	-
VL Day 2 | OP | Subgenomic	0.45 (0.3, 0.58)	0.39 (0.23, 0.52)	0.5 (0.35, 0.62)	0.51 (0.37, 0.63)	0.55 (0.42, 0.66)	0.63 (0.51, 0.73)	0.33 (0.16, 0.48)	0.3 (0.13, 0.45)	0.97 (0.95, 0.98)	-	-	-
VL Day 2 | Nasal | N1	0.55 (0.41, 0.66)	0.47 (0.32, 0.6)	0.71 (0.61, 0.79)	0.75 (0.67, 0.82)	0.66 (0.55, 0.75)	0.59 (0.46, 0.69)	0.45 (0.3, 0.58)	0.41 (0.25, 0.55)	0.48 (0.33, 0.6)	0.54 (0.4, 0.65)	-	-
VL Day 2 | Nasal | Subgenomic	0.55 (0.42, 0.66)	0.48 (0.34, 0.61)	0.71 (0.61, 0.79)	0.79 (0.71, 0.85)	0.67 (0.56, 0.75)	0.61 (0.49, 0.71)	0.45 (0.3, 0.58)	0.43 (0.27, 0.56)	0.48 (0.33, 0.6)	0.54 (0.4, 0.65)	0.97 (0.96, 0.98)	-

Table S3. Spearman correlation (with 95% confidence intervals) of dose score with virus-specific antibody, by vaccine.

	Janssen (actual)	Janssen (s=1/2)	Janssen (2 dose)	Moderna	Novavax	Sanofi
MN | Day -14	0.43 (0.09, 0.68)	0.55 (0.25, 0.75)	-0.43 (-0.83, 0.27)	0.81 (0.62, 0.91)	0.34 (-0.06, 0.64)	0.31 (-0.09, 0.62)
MN | Day 0	0.33 (-0.02, 0.61)	0.46 (0.13, 0.7)	0.37 (-0.34, 0.81)	0.76 (0.53, 0.89)	0.32 (-0.08, 0.63)	0.15 (-0.25, 0.51)
MSD ECL | Day -14	0.53 (0.22, 0.74)	0.64 (0.38, 0.81)	0.43 (-0.28, 0.83)	0.85 (0.69, 0.93)	0.17 (-0.23, 0.52)	0.52 (0.16, 0.75)
MSD ECL | Day 0	0.57 (0.28, 0.77)	0.63 (0.37, 0.8)	0 (-0.63, 0.63)	0.86 (0.71, 0.93)	0.24 (-0.16, 0.58)	0.6 (0.28, 0.8)
PsVNA ID50 | Day -14	0.42 (0.08, 0.67)	0.57 (0.27, 0.76)	0.5 (-0.19, 0.86)	0.8 (0.59, 0.9)	0.17 (-0.23, 0.52)	0.35 (-0.05, 0.65)
PsVNA ID50 | Day 0	0.46 (0.13, 0.7)	0.6 (0.31, 0.78)	0.43 (-0.28, 0.83)	0.74 (0.49, 0.87)	0.28 (-0.12, 0.6)	0.33 (-0.06, 0.64)
PsVNA ID80 | Day -14	0.6 (0.31, 0.78)	0.71 (0.47, 0.85)	0.57 (-0.1, 0.88)	0.81 (0.62, 0.91)	0.21 (-0.2, 0.55)	0.43 (0.05, 0.7)
PsVNA ID80 | Day 0	0.63 (0.37, 0.81)	0.74 (0.52, 0.86)	0.5 (-0.19, 0.86)	0.84 (0.67, 0.93)	0.27 (-0.13, 0.59)	0.39 (0, 0.67)

Table S4. Linear GEE results, dose score (s=1/2) and AUC | BAL | N1.

	**Estimate**	**Lower 95% CI**	**Upper 95% CI**	**p**
Intercept	38.23	35.40	41.06	<0.001
Janssen dose score	-4.28	-6.33	-2.23	0.002
Moderna dose score	-2.33	-5.43	0.77	0.114
Novavax dose score	-5.37	-7.60	-3.13	0.002
Sanofi dose score	-4.13	-6.12	-2.14	0.002

Table S5. Model selection results using single virus-specific antibody to predict viral load.

Virus-Specific Antibody	Viral Load	Model Selected	Adjusted Rsq
MN | Day 0	AUC | BAL | N1	Exponential	0.522
PsVNA ID80 | Day 0	AUC | Nasal | Subgenomic	Linear	0.504
PsVNA ID80 | Day 0	AUC | Nasal | N1	Linear	0.500
MN | Day -14	AUC | Nasal | Subgenomic	Linear	0.494
MN | Day -14	AUC | Nasal | N1	Linear	0.474
MSD ECL | Day 0	AUC | Nasal | N1	Linear	0.472
PsVNA ID80 | Day -14	AUC | Nasal | Subgenomic	Linear	0.472
MN | Day 0	AUC | Nasal | Subgenomic	Linear	0.468
PsVNA ID80 | Day 0	VL Day 2 | Nasal | N1	Linear	0.458
MSD ECL | Day -14	AUC | Nasal | N1	Linear	0.456
MSD ECL | Day 0	AUC | BAL | N1	Linear	0.448
PsVNA ID80 | Day -14	AUC | Nasal | N1	Linear	0.446
PsVNA ID80 | Day -14	VL Day 2 | Nasal | N1	Linear	0.446
MN | Day 0	AUC | Nasal | N1	Linear	0.445
PsVNA ID50 | Day -14	AUC | Nasal | Subgenomic	Linear	0.443
MSD ECL | Day 0	AUC | Nasal | Subgenomic	Linear	0.441
PsVNA ID50 | Day -14	VL Day 2 | Nasal | N1	Linear	0.440
PsVNA ID80 | Day -14	VL Day 2 | Nasal | Subgenomic	Linear	0.439
MN | Day -14	VL Day 2 | Nasal | Subgenomic	Linear	0.438
MSD ECL | Day -14	AUC | Nasal | Subgenomic	Linear	0.434
PsVNA ID80 | Day 0	VL Day 2 | Nasal | Subgenomic	Linear	0.434
MN | Day -14	VL Day 2 | Nasal | N1	Linear	0.431
MN | Day 0	AUC | BAL | Subgenomic	Exponential	0.426
PsVNA ID50 | Day 0	AUC | Nasal | N1	Linear	0.424
PsVNA ID50 | Day -14	AUC | Nasal | N1	Linear	0.423
PsVNA ID50 | Day -14	VL Day 2 | Nasal | Subgenomic	Linear	0.423
MSD ECL | Day -14	AUC | BAL | N1	Linear	0.407
PsVNA ID50 | Day 0	AUC | Nasal | Subgenomic	Linear	0.404
MSD ECL | Day 0	AUC | BAL | Subgenomic	Linear	0.392
MN | Day -14	AUC | BAL | N1	Exponential	0.389
PsVNA ID50 | Day 0	VL Day 2 | Nasal | N1	Linear	0.385
MSD ECL | Day -14	VL Day 2 | Nasal | N1	Linear	0.381
PsVNA ID50 | Day 0	VL Day 2 | Nasal | Subgenomic	Exponential	0.367
MN | Day 0	VL Day 2 | Nasal | N1	Linear	0.366
MN | Day 0	VL Day 2 | Nasal | Subgenomic	Linear	0.365
PsVNA ID80 | Day 0	AUC | BAL | N1	Exponential	0.363
MSD ECL | Day -14	VL Day 2 | Nasal | Subgenomic	Linear	0.360
MSD ECL | Day 0	AUC | OP | N1	Linear	0.357
PsVNA ID80 | Day 0	AUC | OP | N1	Linear	0.355
MSD ECL | Day -14	AUC | OP | N1	Linear	0.343
MSD ECL | Day 0	VL Day 2 | Nasal | N1	Linear	0.343
MSD ECL | Day -14	AUC | BAL | Subgenomic	Linear	0.341
PsVNA ID80 | Day -14	AUC | BAL | N1	Linear	0.335
MN | Day 0	VL Day 2 | BAL | N1	Linear	0.334
MN | Day 0	AUC | OP | N1	Linear	0.330
MSD ECL | Day 0	VL Day 2 | Nasal | Subgenomic	Linear	0.330
PsVNA ID80 | Day -14	AUC | OP | N1	Linear	0.326
MN | Day -14	AUC | OP | N1	Linear	0.324
MN | Day 0	VL Day 2 | BAL | Subgenomic	Linear	0.322

Record locator#UID-2026-03-13-630

Virus-Specific Antibody	Viral Load	Model Selected	Adjusted Rsq
PsVNA ID50 | Day -14	AUC | OP | N1	Linear	0.316
MN | Day -14	AUC | BAL | Subgenomic	Exponential	0.310
PsVNA ID50 | Day -14	AUC | BAL | N1	Linear	0.303
PsVNA ID50 | Day 0	AUC | BAL | N1	Exponential	0.284
PsVNA ID80 | Day -14	VL Day 2 | BAL | Subgenomic	Linear	0.262
PsVNA ID80 | Day 0	AUC | BAL | Subgenomic	Exponential	0.261
PsVNA ID80 | Day 0	VL Day 2 | BAL | N1	Linear	0.260
PsVNA ID80 | Day 0	VL Day 2 | BAL | Subgenomic	Exponential	0.260
PsVNA ID50 | Day -14	VL Day 2 | BAL | N1	Linear	0.259
MSD ECL | Day 0	VL Day 2 | BAL | Subgenomic	Linear	0.259
PsVNA ID50 | Day -14	VL Day 2 | BAL | Subgenomic	Linear	0.259
MN | Day -14	VL Day 2 | BAL | Subgenomic	Linear	0.257
PsVNA ID80 | Day -14	VL Day 2 | BAL | N1	Linear	0.253
PsVNA ID50 | Day 0	AUC | OP | N1	Linear	0.249
PsVNA ID80 | Day -14	AUC | BAL | Subgenomic	Exponential	0.247
MSD ECL | Day 0	AUC | OP | Subgenomic	Linear	0.247
MSD ECL | Day 0	VL Day 2 | BAL | N1	Linear	0.244
MN | Day -14	VL Day 2 | BAL | N1	Linear	0.241
MSD ECL | Day -14	VL Day 2 | BAL | Subgenomic	Linear	0.237
PsVNA ID80 | Day 0	AUC | OP | Subgenomic	Linear	0.235
MSD ECL | Day -14	AUC | OP | Subgenomic	Linear	0.231
MN | Day -14	AUC | OP | Subgenomic	Linear	0.230
MSD ECL | Day -14	VL Day 2 | BAL | N1	Linear	0.228
PsVNA ID80 | Day -14	AUC | OP | Subgenomic	Linear	0.226
PsVNA ID50 | Day -14	AUC | OP | Subgenomic	Linear	0.222
MN | Day 0	AUC | OP | Subgenomic	Linear	0.211
PsVNA ID50 | Day 0	VL Day 2 | BAL | Subgenomic	Exponential	0.208
PsVNA ID50 | Day -14	AUC | BAL | Subgenomic	Exponential	0.205
PsVNA ID50 | Day 0	VL Day 2 | BAL | N1	Exponential	0.199
PsVNA ID80 | Day 0	VL Day 2 | OP | Subgenomic	Linear	0.196
PsVNA ID80 | Day -14	VL Day 2 | OP | Subgenomic	Linear	0.194
MN | Day -14	VL Day 2 | OP | Subgenomic	Linear	0.186
PsVNA ID50 | Day -14	VL Day 2 | OP | Subgenomic	Linear	0.182
PsVNA ID50 | Day 0	AUC | BAL | Subgenomic	Exponential	0.180
PsVNA ID50 | Day 0	VL Day 2 | OP | Subgenomic	Linear	0.167
PsVNA ID80 | Day 0	VL Day 2 | OP | N1	Linear	0.158
MN | Day -14	VL Day 2 | OP | N1	Linear	0.157
PsVNA ID80 | Day -14	VL Day 2 | OP | N1	Linear	0.155
PsVNA ID50 | Day -14	VL Day 2 | OP | N1	Linear	0.143
PsVNA ID50 | Day 0	AUC | OP | Subgenomic	Linear	0.138
MSD ECL | Day -14	VL Day 2 | OP | Subgenomic	Linear	0.129
MSD ECL | Day 0	VL Day 2 | OP | Subgenomic	Linear	0.128
PsVNA ID50 | Day 0	VL Day 2 | OP | N1	Linear	0.124
MN | Day 0	VL Day 2 | OP | Subgenomic	Linear	0.120
MSD ECL | Day 0	VL Day 2 | OP | N1	Linear	0.112
MSD ECL | Day -14	VL Day 2 | OP | N1	Linear	0.110
MN | Day 0	VL Day 2 | OP | N1	Linear	0.101

Table S6. Percent Variance Explained (with 95% confidence intervals) comparing base model to base + dose + vaccine model, where dose score (s=1/2) is continuous.

	MN | Day -14	MN | Day 0	MSD ECL | Day -14	MSD ECL | Day 0	PsVNA ID50 | Day -14	PsVNA ID50| Day 0	PsVNA ID80 | Day -14	PsVNA ID80 | Day 0
AUC | BAL | N1	88.8 (81.23, 100)	98.2 (96.63, 100)	77.71 (62.88, 96.09)	85.42 (76.65, 100)	71.53 (62.09, 94.64)	71.43 (60.72, 98.51)	75.08 (66.23, 100)	85.9 (79.74, 100)
AUC | BAL | Subgenomic	91.92 (85.6, 100)	97.25 (93.52, 100)	80.09 (63.3, 100)	89.19 (77.58, 100)	67.32 (56.23, 100)	63.02 (49.37, 100)	72.22 (60.1, 100)	83.68 (73.51, 100)
AUC | Nasal | N1	77.44 (67.5, 91.85)	78.51 (69.86, 95.34)	72.73 (62.82, 86.61)	74.44 (65.03, 88.82)	74.32 (64.72, 90.73)	74.52 (65.38, 91.74)	76.64 (67.79, 91.24)	84.57 (76.9, 98.19)
AUC | Nasal | Subgenomic	88.38 (82.98, 99.07)	85.29 (79.61, 99.33)	73.88 (62.91, 90.36)	76.66 (66.94, 91.73)	80.07 (73.37, 94.77)	77.34 (67.72, 93.07)	81.68 (74.48, 92.93)	90.64 (86.61, 99.56)
AUC | OP | N1	82.53 (72.82, 100)	92.28 (86.48, 100)	76.81 (62.11, 94.82)	83.08 (71.26, 100)	76.46 (69.89, 95.33)	71.1 (54.25, 94.11)	83.63 (79, 100)	90.88 (86.33, 100)
AUC | OP | Subgenomic	82.55 (70.55, 100)	77.24 (44.5, 100)	71.52 (47.11, 94.7)	79.33 (58.84, 100)	70.62 (59.3, 98.01)	50.29 (13.47, 80.26)	78.56 (69.63, 100)	82.46 (73.29, 100)
VL Day 2 | BAL | N1	83.29 (75.83, 100)	98.13 (97.55, 100)	65.1 (51.39, 91.13)	73.82 (65.47,97.18)	90.1 (87.76, 100)	84.45 (81.57, 100)	88.23 (84.16, 100)	92.5 (91.65, 100)
VL Day 2 | BAL | Subgenomic	86.67 (80.05, 100)	100 (98.36, 100)	64.54 (48.56, 91.14)	70.41 (56.83,100)	90.11 (84.84, 100)	87.88 (82.15, 100)	89.44 (82.98, 100)	89.96 (83.45, 100)
VL Day 2 OP | N1	74.66 (65.32, 100)	69.86 (59.88, 100)	60.18 (43.55, 100)	51.88 (33.83,100)	76.82 (65.84, 100)	74.18 (62.21, 100)	82.62 (75.78, 100)	92.8 (85.98, 100)
VL Day 2 | OP | Subgenomic	77.99 (66.4, 100)	78.61 (71.45, 100)	68.61 (51.21, 100)	59.14 (39.53, 100)	88.5 (84.59, 100)	84.97 (80.16, 100)	88.99 (84.86, 100)	100 (97.59, 100)
VL Day 2 | Nasal | N1	99.78 (94.37, 100)	100 (98.13, 100)	81.02 (73.62, 100)	78.98 (69.81, 100)	100 (98.32, 100)	96.45 (93.61, 100)	100 (95.88, 100)	100 (93.21, 100)
VL Day 2 | Nasal | Subgenomic	97.53 (93.73, 100)	99.21 (97.65, 100)	81.3 (76.45, 100)	78.89 (72.7, 100)	97.15 (96.18, 100)	93.08 (88.54, 100)	99.74 (99.11, 100)	100 (96.06, 100)

Table S7. RMAC (with 95% confidence intervals) with Sanofi vaccine excluded.

	MN | Day -14	MN | Day 0	MSD ECL | Day -14	MSD ECL | Day 0	PsVNA ID50 | Day -14	PsVNA ID50| Day 0	PsVNA ID80 | Day -14	PsVNA ID80 | Day 0
AUC | BAL | N1	0.44 (0.12, 0.7)	0.59 (0.3, 0.78)	0.18 (-0.05, 0.42)	0.22 (-0.04, 0.47)	0.28 (0.03, 0.52)	0.36 (0.12, 0.56)	0.32 (0.05, 0.53)	0.37 (0.08, 0.63)
AUC | BAL | Subgenomic	0.3 (-0.06, 0.57)	0.48 (0.12, 0.71)	0.14 (-0.11, 0.35)	0.18 (-0.09, 0.4)	0.18 (-0.07, 0.36)	0.19 (-0.05, 0.38)	0.21 (-0.07, 0.43)	0.24 (-0.05, 0.46)
AUC | Nasal | N1	0.52 (0.18, 0.74)	0.6 (0.34, 0.75)	0.34 (-0.07, 0.62)	0.39 (0.02, 0.64)	0.53 (0.2, 0.73)	0.46 (0.03, 0.74)	0.47 (0.16, 0.68)	0.59 (0.26, 0.79)
AUC | Nasal | Subgenomic	0.42 (0.06, 0.72)	0.54 (0.26, 0.76)	0.18 (-0.16, 0.52)	0.22 (-0.13, 0.54)	0.39 (0.08, 0.69)	0.37 (-0.02, 0.69)	0.35 (0.04, 0.63)	0.44 (0.1, 0.73)
AUC | OP | N1	0.2 (0.02, 0.55)	0.15 (-0.08, 0.59)	0.13 (-0.04, 0.45)	0.15 (-0.03, 0.46)	0.2 (0.03, 0.49)	0.11 (-0.13, 0.58)	0.17 (0, 0.5)	0.22 (0.03, 0.55)
AUC | OP | Subgenomic	0.08 (-0.05, 0.5)	0.03 (-0.12, 0.55)	0.03 (-0.09, 0.37)	0.05 (-0.08, 0.42)	0.09 (-0.04, 0.45)	0 (-0.15, 0.53)	0.06 (-0.06, 0.48)	0.09 (-0.05, 0.5)
VL Day 2 | BAL | N1	0.19 (-0.23, 0.49)	0.31 (-0.02, 0.51)	-0.05 (-0.36, 0.17)	-0.02 (-0.32, 0.2)	0.15 (-0.21, 0.4)	0.07 (-0.29, 0.34)	0.11 (-0.22, 0.36)	0.19 (-0.22, 0.46)
VL Day 2 | BAL | Subgenomic	0.22 (-0.13, 0.47)	0.29 (0.01, 0.49)	0.04 (-0.24, 0.24)	0.06 (-0.22, 0.27)	0.16 (-0.14, 0.38)	0.11 (-0.21, 0.35)	0.16 (-0.13, 0.37)	0.19 (-0.12, 0.41)
VL Day 2 | OP | N1	0.3 (-0.06, 0.5)	0.09 (-0.21, 0.27)	0.02 (-0.35, 0.2)	0.06 (-0.29, 0.23)	0.13 (-0.17, 0.3)	0.11 (-0.23, 0.33)	0.17 (-0.17, 0.34)	0.19 (-0.12, 0.37)
VL Day 2 | OP | Subgenomic	0.33 (-0.02, 0.53)	0.14 (-0.18, 0.37)	0.08 (-0.28, 0.29)	0.12 (-0.23, 0.31)	0.19 (-0.14, 0.38)	0.21 (-0.18, 0.46)	0.21 (-0.11, 0.4)	0.24 (-0.09, 0.44)
VL Day 2 | Nasal | N1	0.43 (0.04, 0.67)	0.46 (0.13, 0.64)	0.13 (-0.27, 0.41)	0.19 (-0.18, 0.44)	0.4 (0.02, 0.65)	0.28 (-0.1, 0.56)	0.32 (-0.03, 0.59)	0.49 (0.12, 0.71)
VL Day 2 | Nasal | Subgenomic	0.4 (-0.01, 0.66)	0.42 (0.06, 0.6)	0.06 (-0.36, 0.32)	0.12 (-0.28, 0.38)	0.35 (-0.07, 0.59)	0.23 (-0.18, 0.51)	0.27 (-0.08, 0.53)	0.44 (0.02, 0.67)

Table S8. RMAC (with 95% confidence intervals) with Moderna vaccine excluded.

	MN | Day -14	MN | Day 0	MSD ECL | Day -14	MSD ECL | Day 0	PsVNA ID50 | Day -14	PsVNA ID50| Day 0	PsVNA ID80 | Day -14	PsVNA ID80 | Day 0
AUC | BAL | N1	0.21 (-0.13, 0.48)	0.44 (0.07, 0.7)	0.27 (-0.01, 0.52)	0.41 (0.08, 0.67)	0.16 (-0.13, 0.39)	0.1 (-0.18, 0.33)	0.17 (-0.11, 0.4)	0.2 (-0.11, 0.44)
AUC | BAL | Subgenomic	0.15 (-0.11, 0.37)	0.32 (0.02, 0.53)	0.18 (-0.04, 0.38)	0.32 (0.05, 0.53)	0.08 (-0.14, 0.25)	0.03 (-0.18, 0.2)	0.1 (-0.11, 0.28)	0.11 (-0.12, 0.29)
AUC | Nasal | N1	0.33 (-0.03, 0.57)	0.28 (-0.06, 0.52)	0.46 (0.11, 0.67)	0.62 (0.31, 0.79)	0.16 (-0.14, 0.36)	0.18 (-0.13, 0.4)	0.22 (-0.07, 0.43)	0.32 (0.03, 0.51)
AUC | Nasal | Subgenomic	0.46 (0.15, 0.69)	0.4 (0.1, 0.61)	0.52 (0.24, 0.72)	0.61 (0.37, 0.77)	0.34 (0.08, 0.5)	0.26 (-0.03, 0.46)	0.4 (0.12, 0.57)	0.43 (0.16, 0.59)
AUC | OP | N1	0.4 (0.05, 0.65)	0.41 (0.06, 0.66)	0.54 (0.17, 0.77)	0.63 (0.3, 0.83)	0.39 (0.04, 0.61)	0.24 (-0.15, 0.54)	0.39 (0.06, 0.63)	0.44 (0.07, 0.69)
AUC | OP | Subgenomic	0.28 (-0.04, 0.52)	0.22 (-0.09, 0.45)	0.37 (0.01, 0.62)	0.48 (0.11, 0.72)	0.27 (-0.04, 0.52)	0.09 (-0.25, 0.39)	0.26 (-0.06, 0.52)	0.27 (-0.09, 0.55)
VL Day 2 | BAL | N1	0.22 (-0.17, 0.46)	0.42 (0.04, 0.64)	0.27 (-0.12, 0.51)	0.34 (-0.12, 0.65)	0.27 (0, 0.45)	0.18 (-0.08, 0.34)	0.26 (-0.01, 0.43)	0.25 (-0.04, 0.44)
VL Day 2 | BAL | Subgenomic	0.3 (-0.07, 0.54)	0.45 (0.1, 0.67)	0.34 (-0.01, 0.57)	0.43 (-0.01, 0.72)	0.34 (0.08, 0.52)	0.24 (-0.04, 0.41)	0.33 (0.05, 0.51)	0.32 (0.03, 0.51)
VL Day 2 | OP | N1	0.04 (-0.35, 0.35)	-0.03 (-0.39, 0.25)	0.06 (-0.37, 0.44)	0.07 (-0.41, 0.5)	-0.02 (-0.38, 0.3)	0.01 (-0.27, 0.23)	-0.01 (-0.36, 0.29)	0.05 (-0.3, 0.35)
VL Day 2 | OP | Subgenomic	0.21 (-0.18, 0.49)	0.14 (-0.25, 0.4)	0.2 (-0.23, 0.51)	0.21 (-0.23, 0.58)	0.13 (-0.25, 0.42)	0.18 (-0.15, 0.41)	0.15 (-0.24, 0.43)	0.22 (-0.15, 0.48)
VL Day 2 | Nasal | N1	0.66 (0.34, 0.84)	0.62 (0.35, 0.78)	0.72 (0.49, 0.84)	0.61 (0.38, 0.76)	0.6 (0.33, 0.78)	0.54 (0.25, 0.72)	0.65 (0.36, 0.82)	0.62 (0.32, 0.81)
VL Day 2 | Nasal | Subgenomic	0.57 (0.28, 0.77)	0.5 (0.18, 0.71)	0.6 (0.36, 0.76)	0.51 (0.23, 0.69)	0.46 (0.13, 0.71)	0.46 (0.11, 0.69)	0.52 (0.24, 0.74)	0.47 (0.11, 0.74)

Record locator#UID-2026-03-13-630

Table S9. RMAC (with 95% confidence intervals) with Janssen vaccine excluded.

	MN | Day -14	MN | Day 0	MSD ECL | Day -14	MSD ECL | Day 0	PsVNA ID50 | Day -14	PsVNA ID50| Day 0	PsVNA ID80 | Day -14	PsVNA ID80| Day 0
AUC | BAL | N1	0.22 (-0.21, 0.57)	0.46 (0.1, 0.72)	-0.06 (-0.42, 0.38)	0.16 (-0.22, 0.53)	-0.05 (-0.36, 0.21)	0.01 (-0.32, 0.3)	0.05 (-0.3, 0.35)	0.17 (-0.2, 0.49)
AUC | BAL | Subgenomic	0.15 (-0.22, 0.45)	0.31 (-0.06, 0.59)	-0.13 (-0.45, 0.26)	0.09 (-0.28, 0.42)	-0.06 (-0.37, 0.21)	-0.04 (-0.35, 0.21)	0.02 (-0.35, 0.33)	0.09 (-0.29, 0.4)
AUC | Nasal | N1	0.12 (-0.25, 0.46)	0.14 (-0.31, 0.53)	-0.03 (-0.35, 0.26)	0.11 (-0.22, 0.38)	-0.14 (-0.44, 0.14)	-0.1 (-0.45, 0.24)	-0.03 (-0.39, 0.29)	0.08 (-0.31, 0.44)
AUC | Nasal | Subgenomic	0.28 (-0.1, 0.56)	0.33 (-0.14, 0.68)	0.02 (-0.26, 0.28)	0.17 (-0.11, 0.44)	-0.11 (-0.39, 0.15)	-0.06 (-0.42, 0.27)	0.04 (-0.29, 0.34)	0.2 (-0.22, 0.51)
AUC | OP | N1	-0.01 (-0.37, 0.36)	0.13 (-0.21, 0.48)	-0.04 (-0.31, 0.21)	0.09 (-0.17, 0.34)	-0.17 (-0.47, 0.07)	-0.06 (-0.38, 0.19)	-0.05 (-0.39, 0.23)	0.1 (-0.26, 0.41)
AUC | OP | Subgenomic	0.01 (-0.29, 0.31)	0.13 (-0.23, 0.44)	-0.1 (-0.34, 0.15)	0.06 (-0.21, 0.3)	-0.24 (-0.53, 0.03)	-0.21 (-0.49, 0.06)	-0.1 (-0.41, 0.17)	-0.04 (-0.38, 0.27)
VL Day 2 | BAL | N1	0.15 (-0.16, 0.38)	0.24 (-0.07, 0.46)	0.01 (-0.29, 0.23)	0.07 (-0.19, 0.23)	0.12 (-0.12, 0.31)	0.11 (-0.11, 0.27)	0.14 (-0.14, 0.36)	0.14 (-0.14, 0.35)
VL Day 2 | BAL | Subgenomic	0.16 (-0.1, 0.37)	0.21 (-0.07, 0.45)	0.01 (-0.26, 0.24)	0.07 (-0.16, 0.25)	0.08 (-0.16, 0.29)	0.12 (-0.09, 0.29)	0.14 (-0.14, 0.36)	0.14 (-0.14, 0.37)
VL Day 2 | OP | N1	0.26 (0.04, 0.41)	0.17 (-0.04, 0.28)	0.21 (0.05, 0.31)	0.23 (0.09, 0.32)	0.12 (-0.07, 0.26)	0.07 (-0.13, 0.21)	0.18 (-0.04, 0.32)	0.16 (-0.03, 0.29)
VL Day 2 | OP | Subgenomic	0.33 (0.11, 0.5)	0.21 (-0.01, 0.36)	0.27 (0.11, 0.39)	0.29 (0.14, 0.4)	0.16 (-0.06, 0.33)	0.1 (-0.12, 0.29)	0.24 (0.02, 0.42)	0.22 (0, 0.39)
VL Day 2 | Nasal | N1	0.5 (0.22, 0.71)	0.36 (0.03, 0.61)	0.35 (0.09, 0.55)	0.29 (0.08, 0.46)	0.34 (0.08, 0.56)	0.33 (0.04, 0.57)	0.44 (0.13, 0.67)	0.43 (0.11, 0.67)
VL Day 2 | Nasal | Subgenomic	0.51 (0.24, 0.7)	0.38 (0.07, 0.59)	0.32 (0.08, 0.5)	0.28 (0.08, 0.42)	0.32 (0.09, 0.54)	0.31 (0.01, 0.59)	0.43 (0.13, 0.64)	0.41 (0.11, 0.64)

Table S10. RMAC (with 95% confidence intervals) with Novavax vaccine excluded.

	MN | Day -14	MN | Day 0	MSD ECL 2 | Day -14	MSD ECL | Day 0	PsVNA ID50 | Day -14	PsVNA ID50 | Day 0	PsVNA ID80 | Day -14	PsVNA ID80 | Day 0
AUC | BAL | N1	-0.22 (-0.6, -0.01)	0.25 (-0.25, 0.49)	-0.14 (-0.63, 0.17)	-0.06 (-0.58, 0.24)	0.06 (-0.45, 0.34)	-0.15 (-0.61, 0.13)	0.03 (-0.45, 0.32)	-0.02 (-0.48, 0.21)
AUC | BAL | Subgenomic	-0.35 (-0.67, -0.08)	0.22 (-0.26, 0.57)	-0.23 (-0.64, 0.13)	-0.06 (-0.51, 0.34)	-0.2 (-0.63, 0.15)	-0.44 (-0.79, -0.13)	-0.16 (-0.58, 0.21)	-0.2 (-0.61, 0.13)
AUC | Nasal | N1	0.02 (-0.67, 0.32)	0.01 (-0.61, 0.29)	0.03 (-0.62, 0.33)	0.09 (-0.6, 0.38)	0.06 (-0.63, 0.36)	0.02 (-0.63, 0.32)	0.08 (-0.63, 0.4)	0.18 (-0.49, 0.43)
AUC | Nasal | Subgenomic	0.15 (-0.66, 0.48)	0.14 (-0.53, 0.44)	0.03 (-0.64, 0.39)	0.08 (-0.61, 0.41)	0.33 (-0.42, 0.6)	0.08 (-0.61, 0.41)	0.35 (-0.47, 0.64)	0.38 (-0.36, 0.6)
AUC | OP | N1	-0.07 (-0.61, 0.26)	-0.04 (-0.59, 0.28)	0 (-0.57, 0.33)	0.06 (-0.55, 0.38)	0.25 (-0.43, 0.55)	-0.15 (-0.67, 0.19)	0.17 (-0.5, 0.51)	0.24 (-0.36, 0.52)
AUC | OP | Subgenomic	-0.27 (-0.71, 0.07)	-0.33 (-0.77, 0.02)	-0.24 (-0.7, 0.1)	-0.15 (-0.66, 0.2)	0 (-0.6, 0.36)	-0.52 (-0.84, -0.23)	-0.08 (-0.67, 0.28)	-0.1 (-0.63, 0.24)
VL Day 2 | BAL | N1	-0.19 (-0.43, -0.02)	0.05 (-0.29, 0.28)	-0.23 (-0.49, -0.01)	-0.2 (-0.47, 0.02)	0.02 (-0.29, 0.25)	-0.18 (-0.45, 0.01)	-0.06 (-0.36, 0.17)	-0.08 (-0.35, 0.11)
VL Day 2 | BAL | Subgenomic	-0.26 (-0.69, -0.02)	0.05 (-0.54, 0.33)	-0.28 (-0.76, 0.04)	-0.22 (-0.74, 0.09)	0.06 (-0.46, 0.31)	-0.23 (-0.73, 0.05)	-0.03 (-0.55, 0.23)	-0.14 (-0.64, 0.11)
VL Day 2 | OP | N1	-0.09 (-0.31, 0.07)	-0.19 (-0.41, -0.03)	-0.15 (-0.39, 0.02)	-0.13 (-0.36, 0.05)	-0.13 (-0.37, 0.05)	-0.18 (-0.39, -0.03)	-0.09 (-0.33, 0.09)	-0.09 (-0.32, 0.08)
VL Day 2 | OP | Subgenomic	-0.08 (-0.47, 0.15)	-0.23 (-0.59, 0.02)	-0.17 (-0.56, 0.08)	-0.14 (-0.52, 0.11)	-0.07 (-0.47, 0.17)	-0.15 (-0.54, 0.08)	-0.03 (-0.45, 0.22)	-0.04 (-0.42, 0.19)
VL Day 2 | Nasal | N1	0.09 (-0.22, 0.34)	0.02 (-0.28, 0.24)	0.09 (-0.19, 0.31)	0.03 (-0.25, 0.26)	0.25 (-0.07, 0.51)	0.1 (-0.18, 0.32)	0.22 (-0.12, 0.5)	0.21 (-0.06, 0.42)
VL Day 2 | Nasal | Subgenomic	0.05 (-0.36, 0.33)	-0.05 (-0.46, 0.22)	0 (-0.38, 0.26)	-0.05 (-0.42, 0.2)	0.21 (-0.22, 0.47)	0 (-0.39, 0.27)	0.19 (-0.25, 0.47)	0.13 (-0.28, 0.39)

Supplementary Files

This is a list of supplementary files associated with this preprint. Click to download.

- CoPSAP6.56.6Final.pdf
- CoPSAP6.2Final.pdf
- SAPReportMain.docx
- CoPSAP6.8Final.pdf
- CoPSAP6.4Final.pdf
- NHPCOPSAPV203Mar2022.docx
- CoPSAP6.4AppendixFinal.pdf
- CoPSAP6.56.6AppendixFinal.pdf
- SAPRCodeFinal.zip
- CoPSAP6.3Final.pdf
- nrreportingsummaryFayetal.pdf
- RS1FLTN.pdf
